# Ultra-thin light-weight laser-induced-graphene (LIG) diffractive optics

**DOI:** 10.1038/s41377-023-01143-0

**Published:** 2023-06-15

**Authors:** Younggeun Lee, Mun Ji Low, Dongwook Yang, Han Ku Nam, Truong-Son Dinh Le, Seung Eon Lee, Hyogeun Han, Seunghwan Kim, Quang Huy Vu, Hongki Yoo, Hyosang Yoon, Joohyung Lee, Suchand Sandeep, Keunwoo Lee, Seung-Woo Kim, Young-Jin Kim

**Affiliations:** 1 Department of Mechanical Engineering, Korea Advanced Institute of Science and Technology (KAIST), Daehak-ro, Yuseong-gu, Daejeon, 34141 Republic of Korea; 2 School of Mechanical and Aerospace Engineering, Nanyang Technological University (NTU), 50 Nanyang Avenue, 639798 Singapore, Singapore; 3 Panasonic Factory Solutions Asia Pacific (PFSAP), 285 Jalan Ahmad Ibrahim, 639931 Singapore, Singapore; 4 Department of Aerospace Engineering, Korea Advanced Institute of Science and Technology (KAIST), Daehak-ro, Yuseong-gu, Daejeon, 34141 Republic of Korea; 5 Department of Mechanical System Design Engineering, Seoul National University of Science and Technology (Seuoltech), 232 Gongneung-ro, Nowon-gu, Seoul, 01811 Republic of Korea; 6 LASER N GRAPN INC., 193 Munji-ro, Yuseong-gu, Daejeon, 34051 Republic of Korea

**Keywords:** Laser material processing, Optical properties and devices

## Abstract

The realization of hybrid optics could be one of the best ways to fulfill the technological requirements of compact, light-weight, and multi-functional optical systems for modern industries. Planar diffractive lens (PDL) such as diffractive lenses, photonsieves, and metasurfaces can be patterned on ultra-thin flexible and stretchable substrates and be conformally attached on top of arbitrarily shaped surfaces. In this review, we introduce recent research works addressed to the design and manufacturing of ultra-thin graphene optics, which will open new markets in compact and light-weight optics for next-generation endoscopic brain imaging, space internet, real-time surface profilometry, and multi-functional mobile phones. To provide higher design flexibility, lower process complexity, and chemical-free process with reasonable investment cost, direct laser writing (DLW) of laser-induced-graphene (LIG) is actively being applied to the patterning of PDL. For realizing the best optical performances in DLW, photon-material interactions have been studied in detail with respect to different laser parameters; the resulting optical characteristics have been evaluated in terms of amplitude and phase. A series of exemplary laser-written 1D and 2D PDL structures have been actively demonstrated with different base materials, and then, the cases are being expanded to plasmonic and holographic structures. The combination of these ultra-thin and light-weight PDL with conventional bulk refractive or reflective optical elements could bring together the advantages of each optical element. By integrating these suggestions, we suggest a way to realize the hybrid PDL to be used in the future micro-electronics surface inspection, biomedical, outer space, and extended reality (XR) industries.

## Abstract (web summary)

We present how direct-laser-writing can be utilized to fabricate ultra-thin light-weight planar diffractive optics with graphene as the base material. (Inset scale bar: 1 mm).

## Graphical abstract

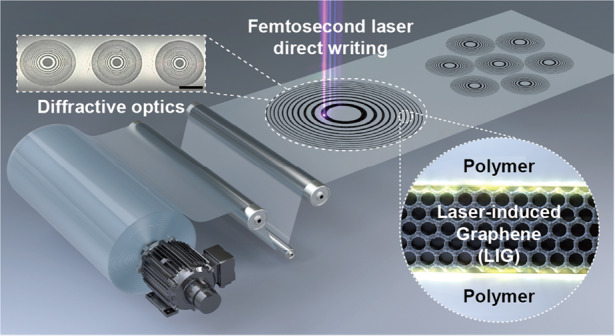

## Introduction

Seeing is believing. Vision is the most dominant capability for us to perceive the surrounding environment. Through vision, we acquire a variety of information, such as the shape, color, and distance of an object. Optics have been utilized to improve this visual perception process and their usage has gradually increased to date^1–6^. However, despite the increase in use, general optical systems are limited in their size, weight, function, and complicated optical alignment requirement. Next-generation hybrid optics will overcome these limitations and provide superior performance compared to the traditional optical system by exploiting the advantages of refractive^6^, reflective^7^, and diffractive^2–5,8^ optics and metasurfaces^9–11^. Traditionally, hybrid optics are fabricated by patterning optical elements on the surface of conventional bulk refractive or reflective optical elements^6,12^. However, the high-resolution manufacturing requirements of diffractive patterns on top of the free-form refractive or reflective optical surfaces have hindered their widespread implementation^8,13^. In the context of integrated hybrid systems, the conventional solution is to stack or align several diffractive and refractive optical elements in series^6,12^. Such combinations are associated with complicated processes with custom-made bulky manufacturing equipment. Moreover, these processes are not sufficiently versatile to work with arbitrary geometries.

**Fig. 1:**
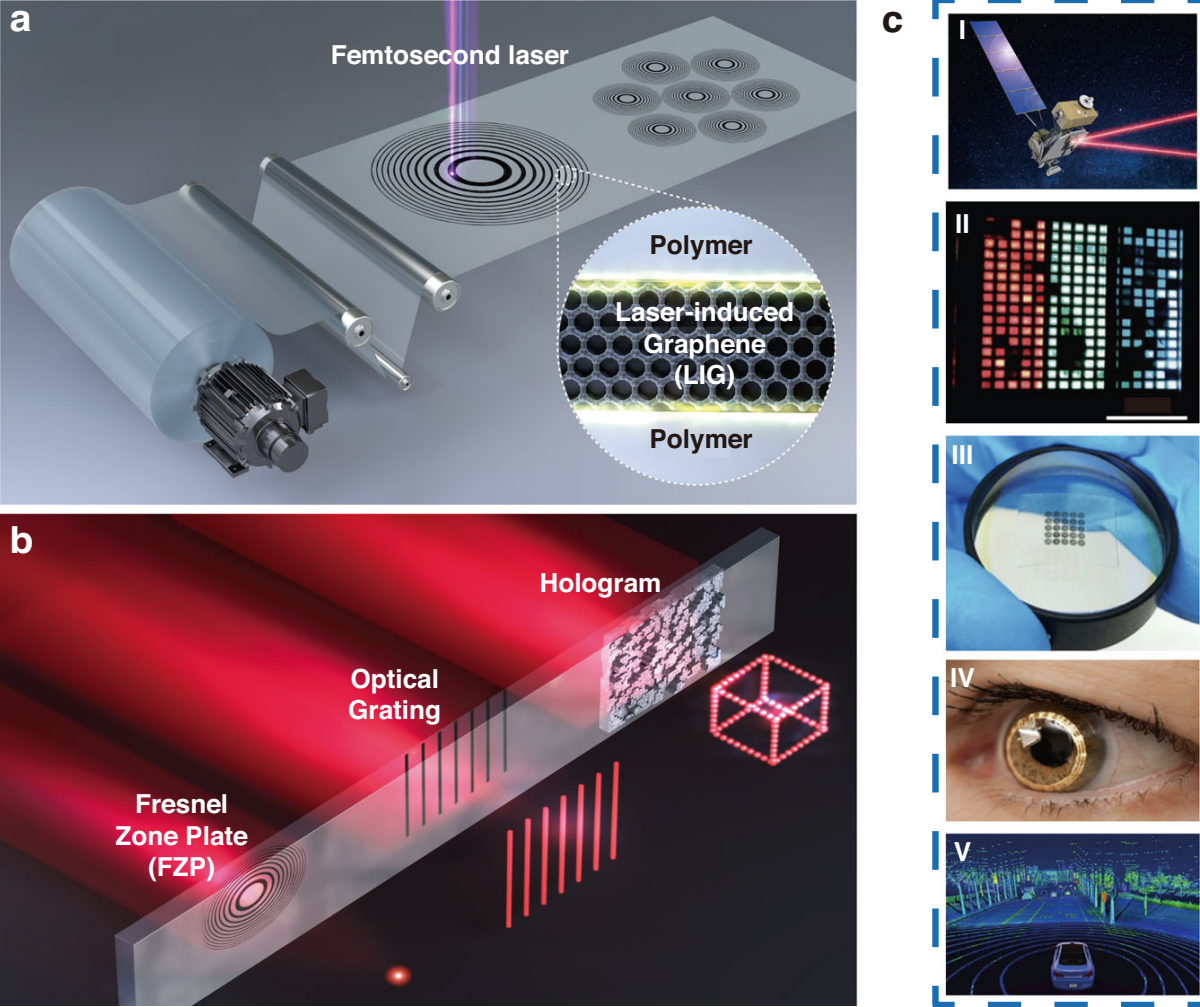
Direct laser writing of PDLs. **a** Schematic diagram of PDL patterning via direct laser writing. This example shows a case of PDL mass production with a roll-to-roll process, which is ready to be applied in various industries. **b** Light propagation image of manufactured ultra-thin PDLs (from left, FZP, grating, and hologram). **c** Future applications of PDLs in aerospace optical communication, flexible display, hybrid optics, bio applications and vehicle sensors (**i** Adapted withpermission from NASA^140^. **ii** Adapted with permission from ref. ^141^., Springer Nature. **iii** Adapted with permission from ref. ^139^, IOP publisher. **iv** Adapted with permission from ref. ^142^, Elsevier. **v** Adapted with permission from ref. ^143^, Elsevier)

By taking the industrial demands on mass production into account, direct laser writing (DLW) of planar diffractive lens (PDL) can be considered as the potential alternative technology for realizing hybrid optics as shown in Fig. 1a, b^8,9,14–17^. PDL, hereafter, includes 1D/2D diffractive optics, Fresnel zone plates, photonsieves and metasurfaces as shown in Fig. 1b. A conformal layer of direct laser-written PDL on top of flexible or stretchable substrates can be directly attached onto the surface of arbitrary optical surfaces^8,9,14,15,17^. This combination offers positive incooperation of the key advantages of each optical component; it could further provide totally new unprecedented functionalities. Novel 2D materials, such as graphene^8,9,14–16,18^, molybdenum disulfide (MoS_2_)^19^, and MXene^20^ can be considered as the base optical material; these could provide new regimes of optical permeability and permittivity as well as the electrical properties for future electrically active adaptive optics. Here, we start with the graphene as the most widely applied optical base^8,9,14–16,18^. The ultra-thin graphene optics could open new vistas in compact and light-weight optics for next-generation endoscopic brain imaging^21–24^, space observation^25–27^, and multi-functional optics as shown in Fig. 1c.

**Fig. 2:**
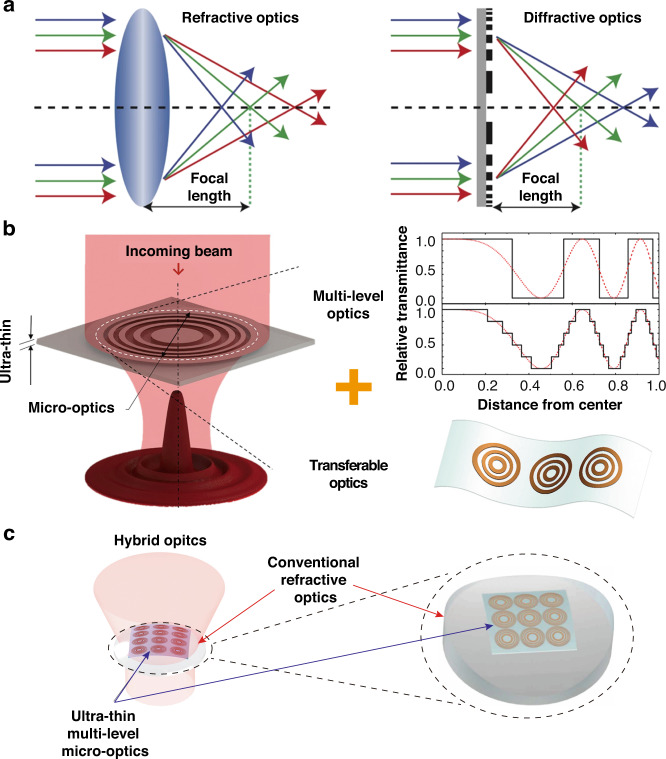
Refractive and diffractive optics for light manipulation. **a** Focusing optics (left: refractive optics, right: difractive optics) of incoming light source at different wavelengths. **b** Schematic of an ultra-thin multi-level micro-optics for focusing light. (insert top right) Thickness profiles for multi-level optics. (insert bottom right) A conceptual diagram of transferred diffractive micro-optics onto a flexible/stretchable substrate. **c** A conformal layer of diffractive optics directly attached to the surface of a conventional refractive lens. **a** Adapted with permission from ref. ^38^, Springer Nature. **b** Adapted with permission from ref. ^8^, Elsevier. **c** Adapted with permission from ref. ^8^, Elsevier, adapted with permission from ref. ^139^, IOP publisher

Regarding spatial array of micro-optics, the refractive micro-optics have long been used in 3D optical imaging based on nature-inspired compound insect eyes^28,29^ Shack–Hartmann wavefront sensors^30,31^, and array confocal microscopes for high-speed biomedical imaging and micro-electronic metal bump inspection^32,33^. Although the fabrication of refractive micro-optics is possible with micro-molding^34^ or lithographic processes^35^, the mass production of the aspheric curved surfaces and special shape features required in refractive optical elements has involved high costs, complicated tools, and long production cycles^36,37^. Converging and diverging refractive optics are based on phase modulation of the incoming light when it passes through the lens material as shown in Fig. 2a. The physical features and material properties of the refractive optics, such as the 3D surface shape, thickness profile and refractive index, determine the light propagation direction. However, the working mechanism of the refractive optics restricts their design flexibility in terms of the lateral size, thickness and weight. Contrarily, diffractive optics can provide significantly higher design flexibility^14,38^. Figure 2a shows the comparative focusing mechanisms of refractive and diffractive optics under different input wavelengths.

Planar diffractive lens (PDL), such as Fresnel zone plates (FZP), are constructed with base unit diffractive elements arranged in the lateral domain, which specifically tailor the beam direction by coherent interference of incoming light. Compared to refractive optics, diffractive optics are relatively thin and can be miniaturized down to the wavelength scale^39–42^. Therefore, they can be excellent alternatives for realizing ultra-thin compact light-weight optical systems. However, the mass-production of such planar diffractive elements requires high-resolution manufacturing, such as photolithography or nano-imprinting on ultra-thin substrates. This requirement is even more stringent for metasurfaces because they require a sub-wavelength patterning resolution with a high aspect ratio to provide novel optical functionalities^40,43–45^. With direct laser writing onto the 2D materials, one could actively manipulate the optical transmittance or optical phase delay at the PDL^8,14,16,46^. With dedicated laser parametric control, multi-step transmittance and phase profile be prepared for higher efficiency beam focusing with suppressed spatial side lobes around the main focal spot, as shown in Fig. 2b. These microscale diffractive optics can provide a relatively smaller refractive index gradient, which can be far improved by incorporating with conventional refractive, reflective or diffractive optics, as shown in Fig. 2c^8^. An optical system generally consists of several optical elements and is configured by aligning or stacking them along the propagation axis. Occasionally, it includes bulky, complex, and custom-made fixtures to align and group the optical elements, which have non-flat surface profiles. Hence, a sophisticated optical configuration results in high integration costs and excess weight^2,47^. The concept of stacking multiple conformal optics attracts considerable attention recently. This approach involves the construction of ultra-thin multiple layers of optical elements that can be directly attached to the surface of an arbitrary object, then, stacking them in a simple way could provide the novel optical functionalities with minimal complexity and weight^43^ (Fig. 2c).

In this review, we introduce recent research efforts addressed to design and manufacturing to realize the widespread industrial use of ultra-thin LIG hybrid optics, which will open new markets in compact and light-weight optics for future endoscopic brain imaging, high-speed space internet, and multi-functional mobile phones. The concept of combining ultra-thin light-weight diffractive optics with other optics (such as refractive and reflective) will be the initial starting point, which could bring together the advantages of each optical element^8^. As illustrated in Fig. 2b, c, ultra-thin diffractive optics with tailored optical properties can be subsequently transferred to flexible/stretchable substrates to serve as conformal layer optics for integration with other optical components. For higher design flexibility, lower process complexity, and chemical-free process with reasonable investment cost, direct laser writing (DLW) of laser-induced-graphene (LIG) is actively being applied to the patterning of PDL. For realizing the best optical performances in DLW, photon-material interactions have been studied in detail with respect to different laser parameters^14,48–51^; the resulting optical characteristics have been evaluated in terms of amplitude and phase^14^. A series of exemplary laser-written 1D and 2D PDL structures have been demonstrated with different base materials and the cases are expanded to plasmonic and holographic structures^9,11,52^. The combination of these ultra-thin light-weight PDL with conventional bulk refractive or reflective optical elements could bring together the advantages of each optical element.

Firstly, we introduce micro-patterning technologies available for realizing ultra-thin PDL^3,14,16,17,39–42,44–46,53^. Over the traditional methods, we consider direct laser writing (DLW) as a promising solution candidate. Various patterning methods have been reported with different energy sources^16,48,50,54^; the laser-based couterpart also has similar system components: laser, beam expander, galvano scanner, f-theta lens, and sample stage. Secondly, graphene-related optical base materials are introduced: graphene, reduced graphene oxide (rGO), and laser-induced graphene (LIG). Detailed material characteristics, including chemical^55–57^, electrical^58–62^, mechanical^59,63–66^, and optical^64,67–70^ ones, are analyzed in detail. Then, photon-material interactions with different key laser parameters are discussed for realizing the optimal PDL performances. Thirdly, the optical performances of the patterned PDL are characterized by comparing the design, simulation, and experimental performances. The characterization starts with the simplest 1D/2D gratings and Fresnel zone plate (FZP); then, expands to an FZP array transferred to a convex refractive lens further to plasmonic and holographic samples^3,7–9,11,14,15,52,53,71,72^. Although these series of examples could not approach hybrid optics readily, the forthcoming efforts will make successful hybrid PDL cases in the near future. Fourthly, the promising applications of graphene-based PDL are introduced in endoscopic bio-imaging^21–24^, lightweight space optics^73^, fast surface profilometry^33^, and complex functional hybrid optics in extended reality (XR) industries^74^. Finally, we would like to present our foresight on what kind of follow-up research works should be accompanied in the future for mass-production ultra-thin light-weight PDL for wider-spread industrial applications.

## Planar diffractive lens: patterning technologies

### Patterning strategies for various planar diffractive lens: summary

**Fig. 3:**
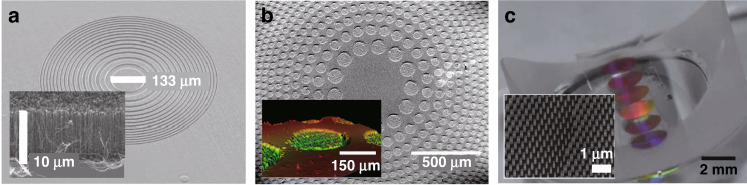
Planar diffractive optics. Planar diffractive lenses of (**a**) zone-plate, (**b**) photon-sieves, and (**c**) metasurfaces. **a** Adapted with permission from ref. ^53^, Springer Nature. **b** Adapted with permission from ref. ^17^, The Optical Society. **c** Adapted with permission from ref. ^43^, Springer Nature

Recently, a conformal layer of metasurfaces intended for arbitrarily shaped multifunctional optics was demonstrated in Refs. ^9,11,40,43–45,52^. The authors present three main requirements for constructing the conformal layer of the PDL. Firstly, the device must be sufficiently flexible to conform to general curved surfaces with a small bending radius in one or more directions. Secondly, the device should function according to optical design requirements. Thirdly, a flexible manufacturing process is the prerequisite to pattern arbitrarily shaped PDLs. These impose unique design and manufacturing constraints: ultra-thin optical structures, flexible substrates, and a simple integration process. In order to meet these constraints, new breakthroughs should be explored through a combination of emerging nano-materials with flexible and stretchable polymeric substrates, and novel manufacturing technologies. PDLs realize the focusing of light by tailoring the interference of light diffracted from all the PDL segments by optimizing their relative amplitudes and phase delays^3,14,16,17,39–42,44–46,53^. Based on their structure, PDLs can be classified into zone-plate, photon-sieve, and metasurfaces, as shown in Fig. 3^17,43,53^.

First, Fresnel zone plates consist of alternating transparent and opaque zones to achieve both amplitude and phase modulation for wavefront shaping. This approach has long been implemented on various materials such as silica^72^, aluminum film^39^, graphene^8,14,16,18^, gold^46^, and nickel^46^. Although there have been numerous demonstrations of the Fresnel zone plates, there have been limited reports still on flexible lenses. Li et al.^53^ presented a flexible PDL with vertically aligned carbon nanotubes (CNTs) percolated into polydimethylsiloxane (PDMS) so as to achieve stretchable amplitude-based zone-plate lens. This work combined the excellent optical absorption of CNT with high transparency and stretchability of PDMS for active control of focal point (Fig. 3a). Similarly, *Moghimi* et al.^3^ used silica nanowires with PDMS to construct an array of micro-FZPs for wide field-of-view imaging^3^. However, the incident light was partially reflected or absorbed by opaque zones in both approaches, which drastically reduced the focusing efficiency of the PDLs. The theoretical focusing efficiency of the amplitude-based zone plate was approximately 10%^4^. Instead of blocking all the opaque rings, Rayleigh proposed the concept of the phase-reversal zone plate. The phase-reversal zone plate utilizes the engineered optical thickness or refractive index of each ring to make a phase delay. Ideally, the phase profile should vary gradually over a zone and return to π at the starting point of the next zone^75^. Such a transparent zone plate can translate into an irradiance increase by a factor of four. Fabrizio et al. used such a phase modulation on a nickel plate and achieved a maximum efficiency of 55% and a reduction in unwanted diffraction orders^46^. Similarly, GO was used to create a phase modulation zone plate to facilitate broad wavelength operation and a focusing efficiency of more than 32%^16^.

Second, the photon-sieve lens evolves from the zone plate-based lens, with the transparent zone replaced with non-overlapping, different-sized nanoapertures. The spatial phase profile is acquired accumulatively from the waves propagating through the plasmonic or photonic waveguide modes, supported by nanoapertures. By properly designing the nano-aperture position, the side lobes of the focal point can be adequately suppressed and the transmitted wavefront can be shaped into a chosen intensity distribution^71^. Similar to zone plate-based lenses, the amplitude photon sieve has inherently lower diffraction efficiencies; hence, transparent phase modulation concepts are introduced to increase the optical throughput reported for phase modulation photon sieve lenses with high flexibility, as shown in Fig. 3b to achieve diffraction efficiencies as high as 49.7%^17,76^.

Third, metasurfaces consist of sub-wavelength-sized building blocks, such as TiO2, SiO2, gold, and silver, which are appropriately adjusted in terms of size, orientation, geometry, and arrangement to control and modify the incident light in phase, amplitude, wavelength, and polarization^35^. Using TiO_2_, efficiencies as high as 66–86% have been achieved for a lens with a numerical aperture (NA) of 0.8^77^. Recently, flexible metasurface-based lenses have been demonstrated by a combination of a periodic array of nano posts of amorphous silicon embedded in PDMS, as shown in Fig. 3c. The physically thin and flexible metasurface structure allows for easy conformance to any object surfaces. Conceptually, they can be designed to work in line with other optical elements to endow additional optical functions^43^. Although metasurface-based lenses exhibit many advantages, they require complex manufacturing processes. Table 1 summarizes representative papers on rigid and flexible PDLs^3,14,16,17,39–42,44–46,53^, categorized by lens type, physical features, optical performance, base materials, and manufacturing methods.

**Table 1:** A summary of various PDLs based on different lens type

Lens Type		Form factor	Image	Thickness	Pattern Size	Process	Materials	Wavelength	Focal length	FWHM	NA	Efficiency
Zone-Plate	Amplitude	Rigid^39^		100 nm	40 μm	Ion-Beam Milling	Aluminum	640 nm	10.3 μm	185 nm	1.34	Not Reported
		Flexible^3^		100 nm	450 μm	Lithography	Silica nanowire & PDMS	620 nm	Not Reported	Not Reported	Not Reported	Not Reported
		Flexible^53^		10 μm	∼650 μm	Lithography	Carbon nanotube & PDMS	635 nm	7.00–8.47 mm	Not Reported	Not Reported	Not Reported
	Phase	Rigid^16^		280 nm	∼8 μm	DLW	Graphene Oxide	400–1500 nm	1.56 μm	∼577 nm	Not Reported	>32%
		Rigid^46^		1.3 μm	0.5 mm	EBL	Silicon nitride & Nickel	0.01–10 nm	1 m	300 nm	Not Reported	55%
		Flexible^14^		0.9 μm	656 μm	FsLDW	Graphene Oxide	638 nm	1.39–1.89 mm	12.0 μm	Not Reported	4.5%
Photon- Sieve	Amplitude	Rigid^41^		100 nm	300 nm	EBL	Chromium	633 nm	13.3 μm	∼202.5 nm	0.83	47%
		Rigid^42^		50 nm	652 nm	EBL	Gold	640 nm	11.2 μm	659 nm	0.48	Not Reported
	Phase	Rigid flex^17^		12.5 μm	few mm	DLW	Polyimide	633 nm	10 μm	65.9 μm	Not Reported	49.7%
Meta-surface	Gradient Phase	Rigid^45^		60 nm	200 mm	EBL	Gold	1550 nm	3 mm	200 μm	0.015	1%
		Flexible^43^		720 nm	1 mm	EBL	Amorphous Silica & PDMS	915 nm	3.5 mm	3.5 μm	N/A	56%
	Geometric Phase	Rigid^40^		600 nm	240 μm	EBL	Titanium oxide	405 nm 532 nm 660 nm	90 μm	380 nm 375 nm 450 nm	0.8	86% 73% 66%
		Rigid^44^		100 nm	few mm	Lithography	Aluminum	0.45–0.75 THz	4 mm	Not Reported	Not Reported	Not Reported

The images used in Table 1 were adapted with permission from Springer Nature^3,16,39,41,43,53^, Elsevier^14^, Wiley-VCH^42,44^, The Optical Society^17^, ACS Publications^45^, American Association for the Advancement of Science (AAAS)^40^

*DLW(FsLDW)* Femtosecond laser direct writing, *EBL* electron beam lithography

## Conventional patterning methods for planar diffractive optics

**Fig. 4:**
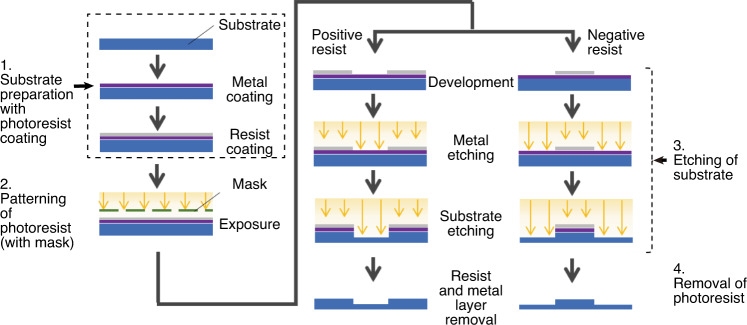
Flowchart for the fabrication of optical structure by lithography

Fabrication methods are an essential aspect of the realization of a PDL product. It determines the cost, function, reliability, and physical features of the product and is closely related to the design and material. Therefore, it is vital to understand the state of the arts, how to fabricate micro-optics and how to integrate the micro-optical elements into flexible substrates. The fabrication of PDLs has been closely related to the microelectronics industry. Although similar, the patterning technologies used to create the physical profiles and microstructures to achieve the required optical properties considerably differ^14^. The patterning methods are categorized into either lithography or direct writing approaches; the majority of PDLs are manufactured via lithography therein. The lithography consists of four stages: substrate preparation with photoresist coating, patterning of the photoresist, etching of the substrate, and removal of the photoresist, as shown in detail in Fig. 4. Sub-micron-resolution PDL nanostructures of PDL have been readily fabricated on materials such as metals and silicon using a lithography process. In most cases, patterned nanostructures are directly used for light manipulation. Thereby, a sub-micrometer resolution down to a few hundred of nanometers is achievable by photolithography (via photons), electron lithography (via electrons), X-ray lithography (via X-ray photons), or ion lithography (via ions)^36^. Although it is evident that high resolution is the key advantage of lithography, this technique is often associated with disadvantages such as high cost, low design flexibility, the need for a pre-designed mask, and the lack of process robustness^17^.

As an alternative, a subset of lithography utilizes the interference phenomenon of light to directly pattern the substrate without the need for a mask. The light source is split into multiple beams and directed to achieve multi-beam interference; coherent multi-beam interference lithography offers the capability to fabricate nanosized periodic structures over a large area. As an example, 123 million arrays of micro-lenses (with a 900-nm period) were fabricated on areas of ~1.0 cm^2^ of flexible polycarbonate sheet in a few seconds under an ambient atmosphere. In summary, this technique is fast, low-cost, and straightforward for producing optical patterns compared to mask-based lithography^78,79^.

Laser is a common energy source used for direct writing. In the case of DLW, the photon can usually be directed to the stationary sample via a set of galvo mirrors and then focused on the plane by an f-theta lens for higher productivity; or the photon can be focused into a smaller spot by an objective lens and the samples can be translated in XYZ domain by mechanical stages. A critical advantage of laser-based systems is their high degree of flexibility. DLW allows arbitrary patterns to be generated on samples without needing a pre-designed mask. In addition, the energy source used to irradiate the samples can easily vary depending on the requirements of the features; the controllable laser parameters include wavelength, power, pulse duration, and repetition rate, while the beam delivery parameters do beam size, scan speed, focal length, and the number of scans. Therefore, DLW does not require any corrosive chemical etching or stringent environmental conditions.

Table 2 illustrates three representative PDL patterning techniques^78,80^, DLW, photolithography, and interference lithography; their key characteristics are compared with respect to resolution, productivity, design flexibility, process complexity, and investment cost. In comparison, DLW is much simpler, highly flexible, and requires much lower implementation costs. The tradeoff to consider is lower productivity owing to the single-point processing technique and relatively lower spatial resolution. Recently, fs (femtosecond: 10^−15 ^s) lasers have been introduced to DLW to achieve finer process control and higher patterning resolution. The fs laser generates a train of repetitive ultra-short light pulses, high peak power with low average power (e.g., 100 fs pulse duration, 1 MW peak power, and 10 mW average power at 100 kHz repetition rate). Therefore, with a femtosecond laser, the nonlinear photochemical process could generate a narrower pattern linewidth than the diffraction-limited focal spot size (~300 nm), even beyond the optical diffraction limit, which is contrary to patterning with conventional continuous-wave (CW) lasers^16^. Alternatively, they exploit the shorter pulse-to-pulse time spacing to limit the heat-affected zone (HAZ)^48,81,82^ The heat diffusion time of the polyimide (in the order of microseconds) is relatively longer than the pulse duration of the fs laser, so heat dissipation to the surrounding volumes could be controlled to be minimal. The fs laser pulses at high repetition rates result in heat accumulation before the heat can be dissipated to the surroundings; therefore, active base temperature control is also possible by the adoption of a high repetition rate fs laser. The ability of fs laser to perform machining with minimum heat propagation leads to the name “cold machining” process. In summary, DLW has emerged as a promising patterning technology for facile and cost-effective single-step manufacturing of PDLs.

**Table 2:** Characteristic of PDL fabrication technique. (a) Direct writing based, (b) interference lithography-based manufacturing, and (c) lithography-based

Description	Laser direct witting		Interference lithography		Lithography	
Conceptual Figures						
Patterning Resolution	Tens of μm (pulse laser) Hundreds of nm (fs laser)	Medium	Hundreds of nm	Medium	Tens of nm	High
Process Complexity	Single step process, ambient environment, tunable laser and optomechanical parameters	Low	Single step process, ambient environment, tunable laser and optomechanical parameters	Medium	Multi-stage process, mask preparation, chemical etching	High
Design Flexibility	Digital pattern easily converted actual	High	Low capability to change of design. Complex optical setup to achieve interference	Low	Pre-mask preparation to convert to design to actual	Medium
Productivity	Single-point processing	Low	Large area processing	High	Large area processing	High
Investment Cost	Small-area facility with low environmental requirement, simple pattering equipment setup	Medium	Small-area facility with low environmental requirement, complex opto-mechanical setup (to cater irregular pattern)	Medium	Multi-equipment setup with high environmental and chemical management	High

### Direct laser writing (DLW) for diffraction optics: system layout

**Fig. 5:**
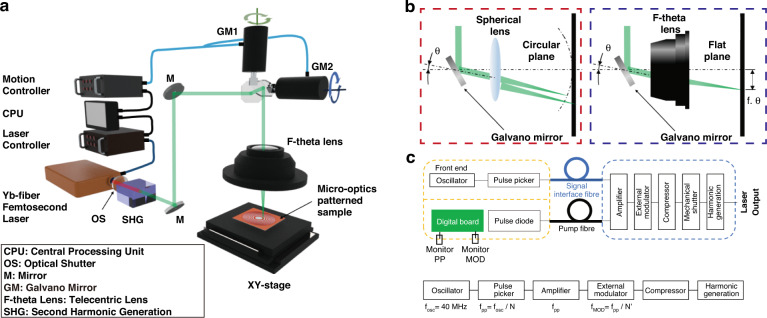
DLW of PDLs: system layout. **a** The system configuration of FsDLW for patterning LIRGO micro-optics through laser and opto-mechanical control. **b** Schematic diagram of galvano mirror with left. spherical lens or right. f-theta lens. **c** Main components for *fs* laser source. **a** Adapted with permission from ref. ^14^, Elsevier

DLW is a patterning technology with high design flexibility that can create arbitrary patterns without pre-prepared masks or a toxic chemical etching process. An example DLW system is illustrated in Fig. 5a^14^. A photo-generating laser, the central part in laser processing, is used as the energy source for the overall DLW system. The wavelength, peak power, and pulse width are the key factors from the laser side; the mirror coatings and lens materials in the DLW system must be carefully selected considering the laser wavelength and damage threshold of the optics. Additional beam control parameters include the beam size, scan speed, focal length, and the number of scans, which can be set in the control unit of the DLW system, consisting of a central processing unit (CPU), laser controller, and motion controller.

According to the patterning strategy, the DLW system is divided into two types. In the first form, the laser beam is raster scanned by the opto-mechanical components, such as a galvano-scanner, acousto-optic beam deflector, or MEMS mirror. In the other form, the laser beam is set stationary point while the 2D or 3D mechanical stage, where the sample is mounted on, is being translated. Various analog and digital signals from sensors are processed in the central unit and used to control a set of DLW parameters. By active control of these key parameters, the optical properties of optical materials, such as graphene can be actively tailored. Therefore, fine parametric control in DLW is critically important in the optical processing of PDL for realizing the designed transmission ratio and phase delay in an efficient manner^14^. This facile control capability of DLW is a significant advantage over its traditional patterning counterparts.

Maintaining the spot size of the laser beam to be the same is important because it determines the patterning resolution over the patterning. however, a simple spherical lens cannot maintain the focal point on the same sample plane due to the existence of spherical aberration, as shown in Fig. 5b-1. Therefore, an f-theta lens or telecentric lens must be used together with a galvano scanner so as to maintain stable processing performances, as shown in Fig. 5b-2. When adjusting the spot size, the lens should be changed to different ones so that the focal spot is formed at the lens’ design focal distance. A similar example is the change of the magnification of the objective lens in microscopes to form a smaller spot size.

Two critical parameters determining the scalability of a manufacturing process are the patterning speed and patterning area. In the case of a Galvano-scanner-based process, the laser beam is reflected by a set of light-weight mirrors, and directed to the target samples; thus, the low-level inertia of these mirrors enables us to realize high-speed continuous processing, such as roll-to-roll process. In addition, the patterning area can be controlled by selecting an appropriate telecentric or f-theta lens to a few to hundreds of millimeters, with the compromise of the patterning resolution. In the case of the patterning process based on the mechanical translation stages, the stage’s relatively large inertia makes the process rather slow, but highly precise. The laser beam is focused on a stationary point by an objective lens without any movement while translating the target sample. By adopting a high magnification objective lens, sub-micrometer-level diffraction-limited patterning is possible over a large patterning area. Therefore, patterning via mechanical stages is well-suited to manufacturing of large-scale optics with fine pattern requirements.

One of the key parameters in laser material processing is the accumulated heat during the processing. Recently, mode-locked fs lasers have been introduced to DLW which provide additional controllability in the accumulated heat. Femtosecond lasers generally deliver ultrashort pulses of less than a few hundred fs to deliver the photon energy at shorter time duration than the required for the heat transfer of ~ps^48,81,82^. This significantly reduces the thermal energy effect on the material, thus prevents unexpected thermal effects in nearby area, and minimizes the heat affected zone (HAZ) in the material^48,81,82^. This allows for narrower pattern linewidths even beyond the optical diffraction limit. Even with fs lasers, some of the photon energy can be converted to heat. Therefore, by shortening the pulse-to-pulse time interval (the inverse of the pulse repetition rate: e.g., high repetition rates of several hundred kHz or higher), the energy transfer rate can be increased. Thus, higher efficiency material processing through heat accumulation is also possible; however, this could also cause thermal-related side effects, similar to those observed with CW lasers; therefore, a dedicated parametric control is a prerequisite. This series of patterning parameters are readily in our hands with a fs laser; an in-depth understanding the laser and beam delivery system should be accompanied by for the optimal patterning, as shown in Fig. 5c.

## Graphene, reduced graphene oxide, and laser-induced graphene

### Graphene-based materials for optical applications

**Fig. 6:**
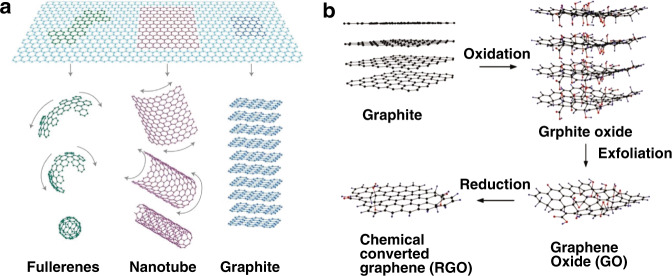
Graphene: basics and synthesis methods. **a** Graphene is a 2D carbon building material with all other dimensionalities. **b** Schematic illustration of the preparation of GO from graphite. **c** Schematic illustration of the structural model of a GO sheet. **a** Adapted with permission from ref. ^101^, Springer Nature. **b** Adapted with permission from ref. ^144^, Elsevier

Graphene is an allotrope of carbon in which six carbon atoms form a monolithic honeycomb unit lattice structure in a single layer^55–57^, as shown in Fig. 6a. In 2004, graphene was successfully isolated and rediscovered by A. Geim and K. Novoselov who were awarded by the Nobel Prize in Physics in 2010 via mechanical exfoliation with adhesive tapes^56^. Graphene has unique electrical, chemical, optical, and mechanical properties, owing to its unique structure; it has a large theoretical specific surface area (2630 m^2^ g^−1^)^55^, high Young’s modulus (~1.0 TPa)^65^, very high carrier mobility (200,000 cm^2^ V^−1^ s^−1^)^83^, high light transmittance (~97.7% at wavelength of 550 nm)^84^, and high thermal conductivity (~5000 Wm^−1^ K^−1^)^85^. Owing to these excellent material properties together with biocompatibility, they have been actively applied to various applications including electronics, sensors, actuators, photonics, optoelectronic devices, mechanical composite materials, and biomedical devices.

A variety of graphene synthesis methods have been studied and the research is being directed to scalable mass-production of graphene for widespread industrial applications. Early studies started with mechanical exfoliation^56,68^, epitaxial growth of silicon carbide^86^, and chemical vapor deposition (CVD) on solid substrates using gaseous hydrocarbons^57,68^. However, these synthesis methods listed above have a common limitation in the production of large-area graphene, which is directly associated with mass production. It is also accompanied by disadvantages such as higher energy consumption and a larger amount of chemical waste. As an alternative, the generation of graphene layers through the photo-reduction of graphene oxide (GO) and DLW of laser-induced graphene (LIG) from carbon-based precursors could be highly promising^48^.

Photo-reduction methods for GO for the efficient mass production of graphene have received considerable attention. GO is a good precursor for the synthesis of rGO, a graphene-like material (Fig. 6b). Ultra-thin films of GO can be deposited on any substrate through processes such as drop casting, spin coating, spray coating, and so on^14,87^. GO is soluble in various solvents, mechanically robust, chemically stable, and compatible with a wide range of organic and inorganic materials. Most importantly, micro-patterning can be established over a large area simply by tuning the laser parameters using a reduction process, such as DLW, to convert the almost transparent GO to opaque rGO with lower transparency (Fig. 6b). GO is usually prepared according to ‘Hummers’ method’, with graphite-based chemical oxidation and subsequent exfoliation in water with the aid of sonication, as shown in Fig. 6b. Exfoliated sheets containing only one or a few layers of carbon atoms, such as graphene, are named GO sheets. To date, the detailed structure of GO is still uncertain because the final structure differs depending on the synthesis method and the degree of oxidation. However, the structural model proposed by Lerf and Klinowski^88^ is widely known for representing single-layer GO sheets. The carbon atom planes of the graphite oxide are heavily decorated with oxygen-containing groups (OCGs), such as hydroxyl groups, epoxy, and carboxyl groups. The OCGs contained in GO make them electrical insulators, which greatly limit their applications, especially in electronics. The thermal or chemical reduction has been applied to GO in the early days in order to remove oxygen-containing groups to achieve graphene-like structures; the resulting material is commonly referred to as ‘rGO’^49^. The reduction using an aqueous solution of hydrazine^89^, hydrogen plasma^49^, and rapid heating over 1000 °C have been reported for GO reduction. However, these thermal and chemical reductions typically involve high-temperature annealing (above 1000 °C) or the use of toxic chemicals, resulting in high-energy waste or environmental contamination. Furthermore, these processes lead directly to poor compatibility with the device manufacturing process, especially for flexible/stretchable substrates.

As an alternative, selective reduction technology of GO using lasers has been introduced, and research works on the laser patterning of electric circuits and devices based on rGO are being actively conducted^55^. It has distinct advantages, such as low cost, high design flexibility, fast conversion process, tunable reduction, and compatibility with flexible/stretchable substrates. Various light sources can be used as energy sources for the photoreduction of GO, but the lasers are the most promising because of their high collimation degree, small focused spot size, and high energy density. Compared to photolithography, chemical synthesis or other processes, this laser-based photo-reduction process is much simpler and more flexible and allows for much lower implementation costs without pre-designed masks, corrosive chemical etching, and stringent environmental conditions. Therefore, this method is applicable to the fabrication of ultra-thin electronic devices such as strain sensors, pressure sensors, electronic skins, supercapacitors, and photodetectors^49^.

One more promising alternative technology is DLW of LIG by irradiating laser beams onto carbon-based precursors. In 2014, Tour et al.^90^ reported on a study of the generation of porous LIG on polyimide (PI) films using a mid-infrared (MIR) CO_2_ laser as the energy source. The LIG generation mechanism is based on the instantaneous temperature rise of carbon precursors to thousands of degrees Celsius by the intense laser beam; this temperature rise induces the dissociation of chemical bonds in the carbon precursors. During this process, carbon molecules, whose chemical bonds are temporarily broken, recombine to form LIG, while gases of various compositions are emitted simultaneously^48^. This phenomenon occurs in a wide range of substances from commercial polymers to natural materials, such as wood and leaves^48^. This phenomenon occurs in a wide range of substances from commercial polymers to natural materials, such as wood and leaves^47^. Furthermore, bread and paper, which are secondary processed products of natural materials, are also rich in carbon, so they were confirmed to be able to utilized for LIG synthesis and applications^91,92^. Although it is not clear whether LIG produced via this process is single-layered pristine graphene or not, it has properties that are similar to those of graphene. At the same time, LIG is a material that could be used in various ways owing to the advantages of its simple and inexpensive synthesis process; LIG does not require additional special chemicals or processes for its synthesis. In addition, LIG can form carbon electrodes through simple laser irradiation on carbon-containing precursor materials for future integrated electro-optical devices^48^. In the case of LIG, the chemical composition differs slightly depending on the precursor materials. It becomes generally porous because of the gas generated in the process^48,59,62,68,93–95^. The porous structure of LIG can provide strong broadband optical absorption similar to the black paint composed of CNT tubes^96^. In addition, LIG’s controlled porosity with the laser parameters enables us to control the optical phase delay, which provides better optical efficiency, functionality, and controllability to LIG optical devices. This porous morphology could open an additional possibility with high value in various semiconductors, electronics, and energy-storage devices due to the potential in additional material doping, functionalities, and better interaction with electolytes^93^.

### Graphene: material/chemical characteristics

Graphene is a material that has attracted attention for its unique chemical stability since its creation. Before the discovery of graphene, it was known that 2D materials were unstable and difficult to exist^97^, but after the discovery of graphene, research on 2D materials exploded. The chemical stability of graphene has been used as a material for supercapacitors and various studies have been conducted^96^. In addition, coating graphene on Surface-enhanced Raman scattering (SERS) has developed into a coating that prevents damage due to oxidation or corrosion^98^. This chemical inertness shows the prospect of graphene as a material that can be utilized in extreme environments.

**Fig. 7:**
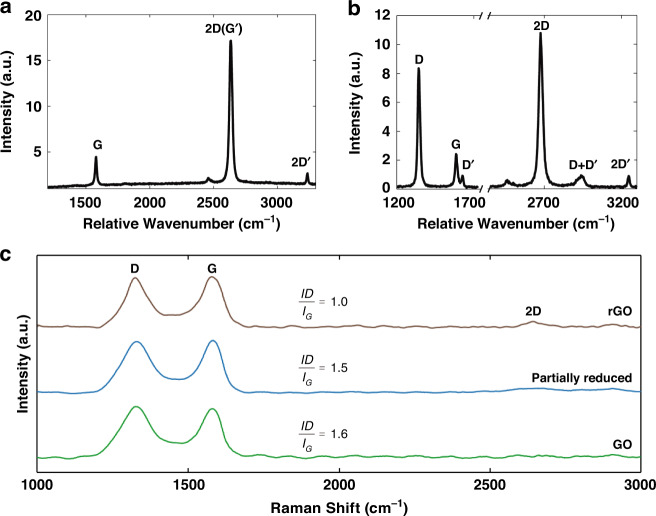
Raman spectrum for characterizing LIG optics. **a** Raman spectrum of a graphene edge, showing the main Raman features, the D, G and G’ bands taken with a laser excitation energy of 2.41 eV. **b** LIG Raman data values on leaves generated by femtosecond laser direct wrting imaging. **c** Raman spectra measurement of GO and photoreduced samples at repetition rate of 500 kHz and a scanning speed of 10 mm s^−1^ for different pulse energies of 20 nJ and 40 nJ. **a**, **b** Adapted with permission from ref. ^100^, IOP publisher. **c** Adapted with permission from ref. ^14^, Elsevier

Various methods have been studied to determine the chemical composition of graphene. Raman spectroscopy is one of the most reliable measurement methods for graphene characterization; it determines the molecular structure by measuring the wavelength-shifted Raman scattering spectra when the sample is excited by a continuous-wave laser beam; the position and strength of Raman peaks contains the information on the bonding status between atoms^99,100^. Raman spectroscopy can be used to determine the number of graphene layers, the degree of internal defects in graphene, the orientation of the graphene sheet, and so on^99,100^. A Raman spectrum of a monolayer graphene (when laser excitation energy of 2.41 eV is used), shows three peaks at 1350 cm^−1^, 1582 cm^−1^, and 2700 cm^−1^. The spectral Raman peaks at 1350 and 1582 cm^−1^ are called D and G peaks, respectively. 2700 cm^−1^ has a G’ band, which is also called a 2D-band, as shown in Fig. 7.

The G band is affected by the sp^2^ carbon-carbon double degeneracy mode within the graphene plane. This region is commonly observed in graphitic materials^62,99,100^. The other major band, G’ (also known as the 2D band), is the 2nd-order Raman peak induced by the breathing mode within the graphene carbocyclic plane^99,100^. In the two aforementioned cases, a Raman signal is generated by carbon bonding and the honeycomb structure of graphene. The D band is caused by phenomena such as non-collinear scattering by phonons^99,100^. This band can enable us to predict the graphene defects because it occurs at the graphene edges or in the regions where the lattice symmetry is empty or broken. By combining these three factors, the composition of graphene can be determined^99,100^.

### Graphene: electrical properties

**Fig. 8:**
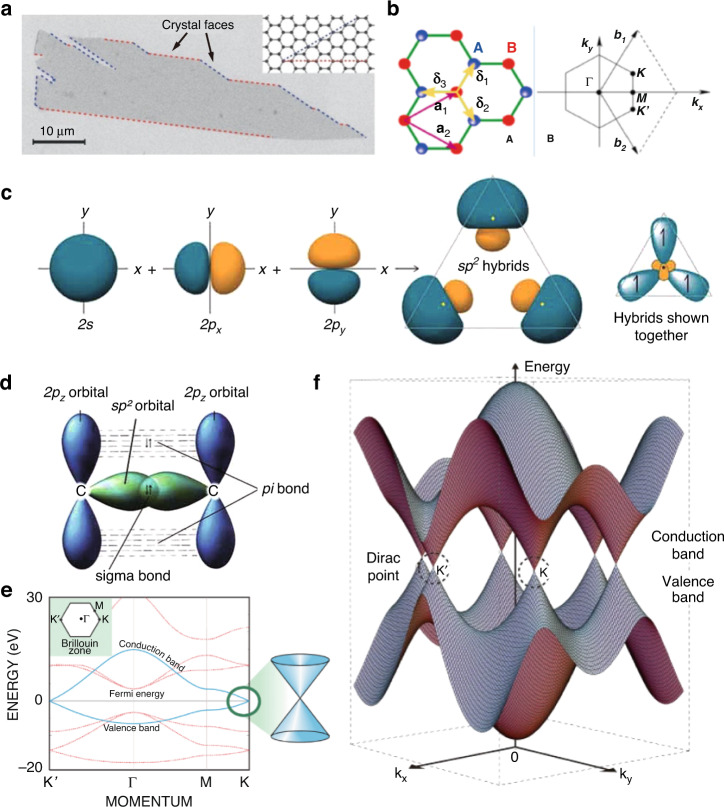
Graphene: electrical properties. **a** Scanning electron micrograph of a relatively large graphene crystal. **b** The honeycomb structure of graphene and Brillouin zone corresponding to each graphene unit. **c** The formation of sp^2^ hybrids. **d** Sigma bond and pi bond formed by sp^2^ hybridization. **e** Energy bands near the Fermi level in graphene. The conduction and valence bands cross at points K and K′. **f** Band structure near the Fermi level of graphene. **a** Adapted with permission from ref. ^101^, Springer Nature. **b** Adapted with permission from ref. ^60^, American Physical Society (APS). Adapted with permission from ref. ^58^, Elsevier. **c**, **d** Adapted with permission from ref. ^64^, Taylor & Francis Group. **e** Adapted with permission from ref. ^97^, American Institute of Physics (AIP). **f** Adapted with permission from ref. ^61^, Springer Nature

Among the advanced materials for the modern semiconductor and electronics industries, graphene has attracted the broadest attention due to its unique electrical properties^58–62^. A typical image of large-sized pristine graphene is presented in Fig. 8a. This material has a unique honeycomb structure created by atomic bonding and exhibits various electrical properties due to electron bias^58,60,61,64^. In a single graphene lattice, carbon lattice structures can be divided into two types, sub-lattices A and B (Fig. 8)^58,61,64,101^. When the carbon atoms share sp^2^ electrons with adjacent carbon atoms under certain conditions, an sp^2^ hybrid bond structure is created, as shown in Fig. 8c. In this planar hexagonal structure, the resulting bond occurs in the atom corresponding to A in Fig. 8b^58^. In another form, this occurs when two carbons are combined in a single-layer graphene structure. In a single layer of pristine graphene (where the graphene unit cells are chemically bonded), the 2p_z_ orbitals of all carbon atoms have sp^2^ orbits perpendicular to the hybridization plane, which forms delocalized π-bonds in the graphene plane of the same layer, as shown in Fig. 8d^64^. In the case of the π-bond caused by this unique structure, free electron movement occurs in the existing plane^58,60^.

The graphene bandgap structure has two interception points and two inequivalent points, K and K’. The diffusion of electrons near these points resembles relativistic Dirac electrons^58,60,62,64,101^; therefore, this point is known as the ‘Dirac point’ (Fig. 8e, f). The valence and conduction bands degenerate at this point, so the graphene is regarded as a zero-gap semiconductor^58,60,62,64,101^. The potential difference between the two ends is perpendicular to the direction of the magnetic field and current; this exhibits unique carriers and excellent electrical properties^58–60,62^. Therefore, the bandgap control of graphene is important in the patterning of electromagnetic devices^58,60,101^. The intrinsic carriers of graphene and massless Dirac–Fermi properties lead to the Hall effect and an unusual quantum Hall effect when electric current flows through a conductor perpendicular to an external magnetic field^58^.

### Graphene: mechanical properties

Due to the strong carbon bonds, graphene provides excellent mechanical properties. However, the measurement of graphene’s mechanical properties is challenging due to its thin cross-section^65,66^. The inelastic properties of 2D materials including graphene are known to be sensitive to internal defects and deformations (e.g., folding, bending, etc.) inside the crystal. The theoretical strength of this material without any defects is presented in Table 3^59,102^.

**Table 3:** Linearly elastic properties of monolayer graphene predicted based on first principles and empirical potential calculations. Adapted with permission from ref. ^59^, Elsevier

Method	2D Young’s modulus Y_2D_ (Nm^−1^)	Poisson’s ratio	Biaxial modulus (N m^−1^)	Bending modulus D_m_ (eV)	Gaussian modulus D_G_(eV)
DFT	345	0.149	406	1.49	–
DFT	348	0.169	419	–	–
DF-TB	–	–	–	1.61	−0.7
DFT	–	–	–	1.44	−1.52
REBO-1	236	0.412	401	0.83	–
REBO-2	243	0.397	403	1.41	–
AIREBO	279	0.357	434	1.56	–
REBO-LB	349	0.132	402	–	–
LCBOPII	343	0.156	406	∼1.1	–

**Fig. 9:**
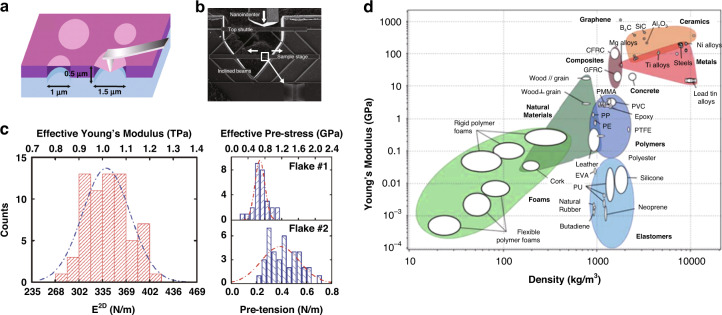
Graphene: mechanical properties. **a** Schematic of nanoindentation on suspended graphene membrane. **b** SEM image showing the results of in situ tensile testing with a microdevice. **c** Elastic response test results. (Left) Histogram of elastic stiffness. (Right) Histogram of film pretensions. **d** Young’s modulus as a function of density for comparing graphene properties to more traditional materials. Note that the axes are in logarithmic scale. Graphene’s density was taken as 2200 kg m^−3^. **a** Adapted with permission from ref. ^65^, American Association for the Advancement of Science (AAAS). **b** Adapted with permission from ref. ^66^, Springer Nature. **c** Adapted with permission from ref. ^65^, American Association for the Advancement of Science (AAAS). **d** Adapted with permission from ref. ^103^, Royal society of chemistry (RSC)

To determine the mechanical properties of graphene, various studies have been conducted^65,66^; Fig. 9a^65,66,103,104^ shows a representative method using an atomic force microscope (AFM). The graphene flake suspended on the Si membrane was pressed by the pointed tip of the AFM cantilever^65^. Similarly, the fracture toughness was measured while increasing the pressure to the tip after placing graphene on a prefabricated suspension microdevice, as shown in Fig. 9b. The nanoindentation test also provided useful data, as shown in Fig. 9c. The resulting mechanical properties can be summarized to Young’s modulus of 1.0 TPa, breaking strength of 130 GPa, and elastic modulus of 0.25 TPa^65^. To compare the properties of graphene with those of traditional materials, Young’s modulus chart based on density is shown in Fig. 9d (both axes are in log scale; graphene density was set to 2200 kg m^**−**3^.) Compared to general metals or ceramics, graphene has a lower density with higher Young’s modulus. Owing to these excellent mechanical properties, graphene can be applied to various mechanical applications where lightweight and mechanical reliability are important^59,63–66^.

### Graphene: optical characteristics

**Fig. 10:**
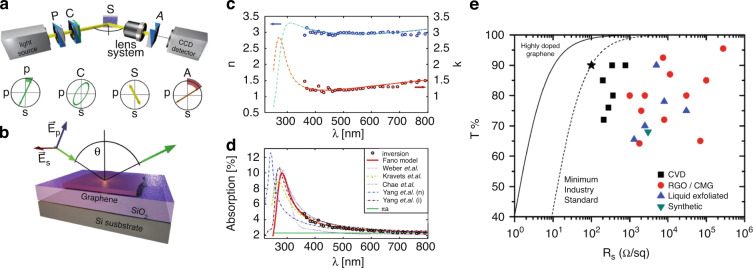
Graphene: optical properties. **a** Schematic representation of the ellipsometric setup; the polarizer, compensator, sample, and analyzer setup (PCSA). Also shown is the state of polarization during null ellipsometry. **b** Optical model of the sample, depicting p- and s-polarizations of light. **c** The complex refractive index of graphene, obtained by inversion (circles), parameterized by the Fano profile (solid lines) and extrapolated Fano model data (dotted–dashed lines). **d** Simulated absorption of a free-standing graphene sheet, based on the data from (**c**). **e** Transmittance and sheet resistance data for papers in the literature. These are broken down into films prepared by CVD, or from rGO or chemically modified graphene, pristine exfoliated graphene, or chemically synthesized graphene. In all cases, the data in the figure correspond to the best data reported. **a**–**d** Adapted with permission from ref. ^69^, American Institute of Physics (AIP). **e** Adapted with permission from ref. ^70^, ACS Publications

Graphene has also attracted attention in flat panel display and transparent electrode industries owing to its unusual electrical conductivity and optical transmittance^64,67–70^. To utilize graphene in display industries, research has been conducted to understand its optical properties^68,105^. Figure 10a, b presents the optical configuration of spectroscopic ellipsometry for measuring the optical properties of graphene in a non-destructive manner^69^. Graphene’s complex reflective index was measured as shown in Fig. 10c^69^. Figure 10d shows the simulation results for graphene’s absorption spectrum in the floating state, which has been presented in a series of papers^69^. General graphene was revealed to have strong absorption in the ultraviolet (UV) region; therefore, transmission-type displays or optical devices at short UV wavelength regimes are not well-suited to graphene. Graphene’s optical properties are also strongly dependent on the patterning method, as presented in Fig. 10e. The pristine graphene (graphene produced by exfoliation), CVD graphene, chemically modified graphene (RGO/CMG), rGO and synthetic growth could provide different levels of transmittance^70^.

During the photoreduction from GO to rGO, the optical properties (transmittance, reflectance, absorptance, and refractive index) significantly change, along with the electrical properties. For example, the absorption coefficient changes from 200% to 300% under the photoreduction over a wide wavelength range from the UV to NIR owing to changes in the surface morphology, interlayer spacing, and chemical composition^106^. The photoreduction leads to a high refractive index modulation (Δn) of ∼0.8, being much larger than that of conventional optical materials; this results in a phase lag of more than π between GO and rGO, even at ultra-thin sub-μm thickness^87^. A thick layer of LIG is opaque in most visible light regions because of the color of the carbonized material and the porous surface morphology. In the case of LIG patterned with a CW laser, the grooves are generated depending on the laser irradiation direction; the formation of such grooves causes optical anisotropy on the surface of the LIG.

### Graphene: photon-material interaction in graphene formation and optical propertie change

The unique advantages of the laser process, such as reliability, conformability, low cost, and design flexibility, and the selective energy delivery of the laser are foundational technologies that have enabled patterning of graphene-based materials. The photon energy of the laser incident on the target carbon precursor is converted into thermal energy through interaction with the precursor material. The resulting thermal energy induces carbonization, exfoliation, photoreduction reactions therein, which are the main causes of the LIG formation and reduction of GO. The energy irradiated to the precursor material generates a high local temperature, which breaks the C = O, C–O, and N–C bonds of the precursor material to induce the carbon rearrangement^48^.

**Fig. 11:**
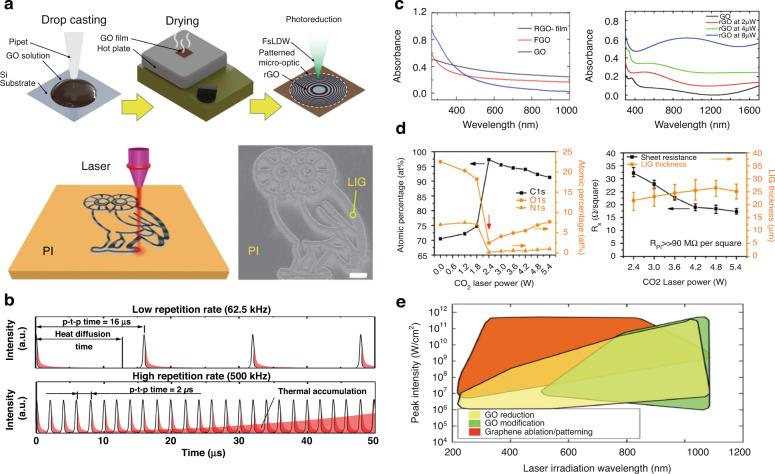
Synthesis of LIG: photon-material interaction. **a** GO film preparation: drop-casting, drying and FsDLW and schematic of the synthesis process of LIG from PI using a CO_2_ laser. **b** Pulse repetition rate effects on heat accumulation in FsDLW. **c** UV–Vis–NIR absorption of few-layered GO (FGO), GO suspension and rGO film. Absorbance of rGO at different laser powers measured using ellipsometry. **d** Atomic percentages of carbon, oxygen and nitrogen as a function of laser power. These values are obtained from high-resolution XPS. The threshold power is 2.4 W, at which conversion from PI to LIG occurs. Correlations of the sheet resistance and LIG film thicknesses with laser power. **e** Distribution of pulsed laser process parameters for graphene ablation, and GO modification over the broad wavelength range of 200–1200 nm. **a** Adapted with permission from ref. ^14^, Elsevier, adapted with permission from ref. ^90^, Springer Nature. **b** Adapted with permission from ref. ^14^, Elsevier. **c** Adapted with permission from ref. ^50^, American Institute of Physics (AIP), adapted with permission from ref. ^16^, Springer Nature. **d** Adapted with permission from ref. ^90^, Springer Nature. **e** Adapted with permission from ref. ^54^, Wiley-VCH

In the photoreduction of GO, various factors must be simultaneously considered. Compared to other reduction methods, photoreduction facilitates the formation of micro-nanostructures and the modulation of the chemical composition simultaneously. GO samples can be exposed to photon energy in numerous ways.^107^ In the case of DLW process, the patterning can be performed by directly irradiating the laser beam onto a GO-coated substrate or the carbon precursor substrate in the atmosphere, as shown in Fig. 11a^14,90^. The laser parameters, such as the operating wavelength, average power, and pulse duration, can be readily tuned (Fig. 11b–e)^14,16,50,54,90^. Therefore, in-depth understanding of the important laser parameters and their impact on the resulting electrical, optical and mechanical performances can provide deeper insight into micro-optical fabrication based on DLW. This section is about the photon-material interaction, which will be split into three sub-sections: wavelength, power, and pulse duration; these will be described in detail in the following subsections.

#### Laser wavelength

The operating wavelength of the laser is an important parameter in laser patterning^16,48,50,54^. Light is reflected, transmitted, or absorbed when a laser strikes the target material. In general laser processing, a wavelength with a high absorption rate must be used with the target material because it is necessary to achieve efficient interaction between the material and incident photons. Using a laser with a high absorption wavelength for the target material results in high efficiency and low energy wastage during processing. The material absorbance versus wavelength for a few-layered GO (FGO) with a thickness of approximately 100 nm to 300 nm and a hydrazine vapor-reduced GO with a thickness of less than 20 nm are presented in Fig. 11c. All three samples exhibited broad absorption bands with pronounced absorption in the UV-region. The absorption peak of GO was observed at 227 nm. Therefore, in the reduction process, the UV light source can be considered to be the light source that most efficiently induces the reduction of rGO. In general, the reduction of GO using laser sources with wavelengths shorter than 390 nm is primarily a photochemical process. It has been reported that the photothermal effect is dominant for GO reduction using lasers with wavelengths longer than 390 nm^49^. The laser reduction must account for both photothermal and photochemical effects owing to the presence of nonlinear effects, including the two- or multiple-photon absorption associated with intense ultrafast laser pulses and other phenomena, such as laser-induced thermal relaxation. In addition to the efficiency of material processing, the operating wavelength determines the focal size, which determines the ultimate patterning resolution. The focused spot size or beam waist *ω*_*o*_ of an input Gaussian beam with beam diameter *D* and wavelength *λ*, can be expressed as *ω*_*o*_ = (*2fλ*)⁄(*πD*) after passing through a telecentric lens with focal length *f*. Given that the focused spot size is proportional to the wavelength owing to the optical diffraction limit, shorter-wavelength laser sources should be considered for high-resolution patterning^108^.

#### Average power and peak intensity

Lasers are the most widely used light sources in PDL patterning. The laser intensity, defined as the laser power per unit area, is closely related to the patterning throughput^48^. Figure 11b shows the correlation between the laser parameters of the pulse laser and the resulting patterning effects^14^. Figure 11c shows the absorbance of few-layer graphene, GO, and rGO. These results show that rGO has a higher light absorption at higher laser intensity levels. The atomic percentage and sheet resistance of LIG patterned on a polyimide (PI) film were measured with regard to the input laser power, as shown in Fig. 11d. In the case of the PI film, a sharp increase in the carbon ratio (∼97%) and a decrease in the oxygen and nitrogen specific gravity were observed. Figure 11e shows the correlation between the laser parameters (peak intensity and wavelength) of the laser and different GO patterning regimes. Although higher peak intensities could elaborate the photoreduction process, it should be noted that the excessive power might result in unexpected ablation or direct removal of the GO^14,54^.

#### Pulse duration

The laser operation mode can be divided into continuous wave (CW) and pulsed modes in the time domain. The energy emission of a CW laser is constant regardless of time. A pulsed laser emits the photon energy at a fixed repetition rate for a set duration. These pulse durations vary from milliseconds to femtoseconds depending on the laser type. When ultra-short laser pulses, less than a few ps, is used for the patterning, LIG or rGO can be patterned with a high spatial resolution with a small heat-affected zone (HAZ)^48,81,82^. Therefore, to increase the spatial resolution of the LIG patterning or rGO formation, ultrafast lasers with a shorter pulse duration is preferred. In addition, the pulse-to-pulse time spacing (the inverse of repetition rate) determines which phenomenon is more dominant from photochemical and photothermal reactions. The correlation between femtosecond pulses and the photothermal response is presented in Fig. 11b.

#### Optical properties of rGO and LIGs

Extensive studies on the electrical properties of LIG and rGO have been made for electronic applications (e.g., various electrical sensors, printed circuit boards, and energy storage devices)^109^; however, there have been limited research works on the optical properties of LIG/rGO. To achieve high optical performances of PDLs made of rGO and LIG, an in-depth understanding of the surface morphology, structural porosity, light transmittance, and phase retardation generated by different laser parameters is essential. In the following section, we describe the actual transmittance measurement data of LIG/rGO obtained based on experiments and the morphology of rGO and LIG surfaces, which are factors that affect optical performance^14^.

#### Parametric studies on the optical characteristics

**Fig. 12:**
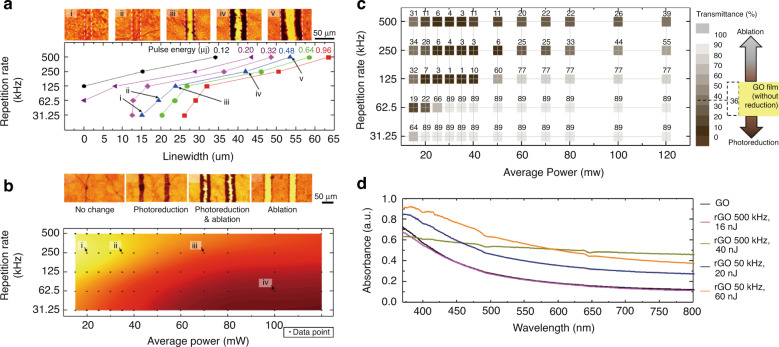
Optical characteristics after FsDLW with different patterning parameters. **a** Linewidth of the patterned GO/rGO with different repetition rates and pulse energies (insert: bright-field optical microscopy images with a pulse energy of 0.48 μJ) and (**b**) processing window diagram with the average power and repetition rate. Optical characteristics after FsDLW with different patterning parameters. **c** Optical transmittance with different patterning parameters. **d** Absorbance spectra of GO and photoreduced samples with different repetition rates and pulse energies using UV-VIS spectroscopy. **a**, **b** Adapted with permission from ref. ^14^, Elsevier. **c** Adapted with permission from ref. ^110^, IEEE Xplore. **d** Adapted with permission from ref. ^14^, Elsevier

The linewidth and transmittance are the most important two output parameters in the patterning of micro PDL. Although laser processing has several key input parameters as presented earlier, the average power, intensity, and pulse interval are generally controlled because the laser wavelength is difficult to change. Figure 12a shows the linewidth and transmittance produced at different repetition rates and pulse energies for the same scan rate of 100 mm s^−1 ^^14^. A gradual increase in linewidth is observed for higher repetition rates and pulse energies. This is because excess energy above the photoreduction threshold causes heat accumulation, which can be viewed as a heat-affected zone (HAZ), resulting in an increase in the linewidth. Figure 12b shows a gradual increase in linewidth from a pristine GO state to a fully ablated state when the repetition rate increases at a constant pulse energy of 0.48 μJ^14^. A similar trend was reported for graphene ablation with increasing laser fluences^51^. Figure 12b shows the photographs of laser-patterned GO after the DLW process at various repetition rates and average powers. The yellow-colored upper left side represents the area where no visual change was observed; the section progressively changes to orange-colored photoreduction regime; a combination of photoreduction and ablation appears as the light brown area; then, finally converges to the ablation regime with the dark brown area at the bottom right side.

Depending on the laser parameters, the transmittance DLW GO can start from 36%, which is the transmittance of the pristine GO, moves up to 89% by rGO formation, and also moves down to 1% by laser ablation, as shown in Fig. 12c^110^. This transmittance can be explained by four cases in conjunction with Fig. 12b. Firstly, when the laser power was lower than the reduction threshold of GO, no photo-reduction occurs. Secondly, when the power was higher than the reduction threshold, a sudden decrease in the transmittance is observed. Thirdly, when the power further increases, the reduction continues and ablation starts to work. Finally, when the ablation process was dominant at the highest power level, both GO and rGO are removed from the sample, resulting in an increase in the transmittance. The absorbance of the rGO was determined by both the pulse energy and pulse repetition rate (Fig. 12d). Based on this in-depth understanding, digital optical patterning at different patterning regimes (phase-changing photo-reduction regime, transmittance-changing photo-reduction regime, and material ablation regime) can be realized.

#### Surface morphology of the photo-reduced GO

**Fig. 13:**
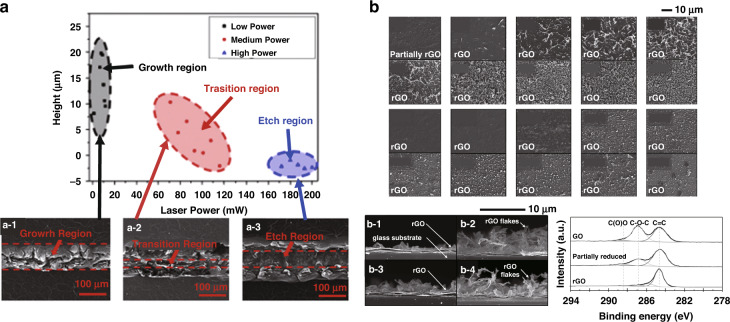
Surface morphology of the rGO. **a** The height profile and surface morphology of a GO film for different average laser power categorised by distinct regimes of (**a-1**) growth, (**a-2**) transition and (**a-3**). **b** Structural analysis of the patterned GO/rGO film at different regimes. Cross-sectional SEM images of GO photoreduction at the selected repetition rate and scanning speed of the sample. C1s XPS spectra of GO, and the rGOs at the repetition rate of 500 kHz, and scanning speed of 10 mm s^**−**1^ for different pulse energies, (**b-3**) 20 nJ and (**b-4**) 40 nJ. **a** Adapted with permission from ref. ^87^, Elsevier. **b** Adapted with permission from ref. ^14^, Elsevier

The degree of conversion (from GO to rGO; carbon precursors to LIG), the thickness of the converted material are important factors in the laser patterning of PDL. The thickness and surface morphology of the material significantly affect its optical properties. The photoreduction of GO can be classified into three regions: the growth region, the transition region, and the etch region. The graphene film thickness decreases with increasing average laser power as shown in Fig. 13a, b^14,87^. At a low power, a relatively large increase in height was observed. The morphology of GO changes into a loosely stacked structure, and the height tends to increase. When the power increases, the etching reaction starts to work and the height gradually decreases. The transition regime shows complex reactions^14,87^. When the laser power exceeds the threshold, it enters the full etching region. In this regime, the oxygen and carbon present in the GO layer are entirely etched to the gas form. Characteristic surface morphologies, Raman spectra, light transmittance, reflectance, and electrical properties are presented in Fig. 13a, b. Therefore, the GO or rGO properties can be simply tuned by changing the key laser parameters.

## Graphene-based ultra-thin flat optics: design and patterning

### Design, patterning, and characterization of Fresnel zone plates (FZPs)

#### FZP patterning and characterization

**Fig. 14:**
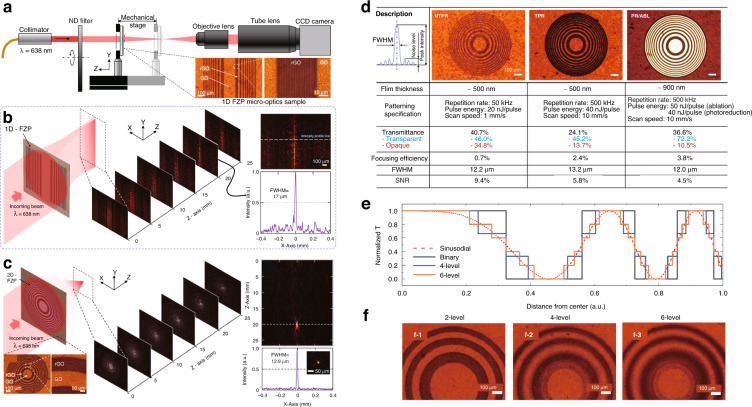
Optical characterization of the 1D GO/rGO FZP. **a** Beam profile measuement system. **b**, **c** Microscopy images of the 1D and 2D FZP. Schematic depicting the focusing of the collimated laser beam by a 1D FZP and the results captured by a CCD camera. **d** List of laser patterning parameters, optical, and mechanical properties of 2D circular FZP patterned using NTPR, TPR, and PR/ABL patterning regimes. Optical characteristic of the ultra-thin diffractive lenses fabricated at three different patterning regimes of NTPR, TPR, and PR/ABL Multi-level FZPs based on tunable photoreduction. **e** FZP design with multi-transmittance levels. **f** Microscopy images of binary, 4-level, and 6-level transparency lens. **a**–**d** Adapted with permission from ref. ^14^, Elsevier. **e**, **f** Adapted with permission from ref. ^8^, Elsevier

Diffractive binary FZPs consist of a set of concentric rings or line with alternative nontransparent zones that diffract the incident light to produce constructive interference at a focal point^3,8,14,53^. Figure 14a shows the operational test experimental setup of the diffraction binary FZP. The measurement results of the fabricated 1D and 2D FZP are shown in Fig. 14b, c. Microscale FZPs are usually manufactured using a lithography process by etching the FZP pattern on a metal film. A new FZP fabrication method involving the photoreduction of rGO from GO using femtosecond laser direct writing (FsLDW) has been recently reported^8,14^. The optical difference was optimized using several laser parameters (average power, repetition rate, and writing speed). For high flexibility and stretchability, the FZP was transferred from the GO/rGO substrate to PDMS films. This facilitates mechanical deformations such as rolling, twisting, and bending, while preserving its optical characteristics. Table 4 provides a summary of related studies on various FZP manufacturing methods and their performances.

**Table 4:** A summary of various FZP fabrication methods and their optical performances

Lens type	Reference	FZP preparation			
		Thickness	Diameter	Process	Materials
**Zone-plate**	Di Fabrizio et al.^46^	1.3 μm	5000 μm	Electron-beam lithography	Silicon nitride & Nickel
	Rogers et al.^39^	0.1 μm	40 μm	Ion-bema milling	Aluminum
	Kong et al.^18^	<0.005 μm	100 μm	Photo lithography	Graphene
	Li et al.^53^	10 μm	Approx. 650 μm	Lithography	Carbon nanotube
	Zheng et al.^16^	0.28 μm	Approx. 8 μm	FsDLW with adaptive optics & 3D stage	Graphene oxide
	Low et al.^14^	0.9 μm	656 μm	FsDLW with Galvano scanner	Graphene oxide

#### Tunable photoreduction of diffractive optics and the resulting optical performance

A binary 2D FZP can be manufactured in three different fabrication regimes^14^: (1) non-thermal photoreduction (NTPR) fabrication, (2) thermal photoreduction (TPR) fabrication, and (3) a combination of photoreduction and ablation (PR/ABL). Using these three regimes, 2D FZPs were fabricated individually with a focal length of 15 mm, and the optical properties are shown in Fig. 14d-1. For the NTPR regime, the repetition rate was fixed at 50 kHz owing to the lower thermal effect of the photochemical reduction. The lower heat accumulation resulted in a low penetration depth of rGO into the GO thin film (Fig. 14d-2). In the TPR regime, the repetition rate was 500 kHz for the combination of photochemical and photothermal reduction, resulting in a high penetration depth of the rGO layers (Fig. 14d-3). Finally, the repetition rate of the fs laser was optimized to simultaneously cause photoreduction and ablation. The z-axis and FWHM data were acquired using the system shown in Fig. 14e. For the case of transmittance, the NTPR regime exhibited the highest value of 40.7%, followed by PR/ABL and TPR regimes with values of 36.6% and 24.1%, respectively. The transmittance differences between GO and rGO were evaluated at 61.7%, 31.5%, and 11.2% for PR/ABL, TPR, and NTPR, respectively. Three representative optical performances (focusing efficiency, focal spot size, and signal-to-noise ratio (SNR)) are evaluated in Fig. 14f. The spot size (FWHM) was evaluated at 12.2 μm, 13.2 μm, and 12.0 μm for the NTPR, TPR, and PR/ABL, respectively. The depth of focus (DOF) was recorded at 1.39 mm, 1.89 mm, and 1.77 mm for the NTPR, TPR, and PR/ABL, respectively. The shortest DOF of the NTPR regime was induced by a low transmission contrast. In addition, the peak intensity was 48% (NTPR) and 78% (TPR) of the PR/ABL regime. In summary, PR/ABL exhibits the smallest focal size, widest DOF, and highest focusing efficiency. This is mainly owing to the high ratio of the transmittance difference between the patterned and ablated areas. A plain FZP has the form of a binary layer, in which light diffracts around the border of an opaque region, causing the diffracted light at the focal point to interfere constructively. Therefore, it is designed to have opaque and transparent rings. The binary form of FZPs reduces design flexibility because there are only two design parameters: blocking or transmitting light. In this respect, rGO photoreduction using a laser is potentially a promising countermeasure for micro-optics that can realize multi-step optical properties, such as phase and transmittance, without a complex photolithography process. Figure 14e presents the transformation of a multi-level FZP into a stepped shape with multiple steps, corresponding to a continuous sinusoidal profile for fabrication. The gradual control of optical transmittance is important in multi-level FZPs, as the transmittance step increases and transitions to a sine wave with a suitable shape. Figure 14f shows the photos of the patterned FZPs for different transmittance steps. We can confirm that the ultra-thin micro-diffraction lens with multi-level transmittance or phase profile has an optical performance similar to that of conventional refractive optical devices. This shows that this technology is a potential alternative to traditional refractive optics.

### Patterning of flexible/stretchable 1D graphene diffraction gratings

**Fig. 15:**
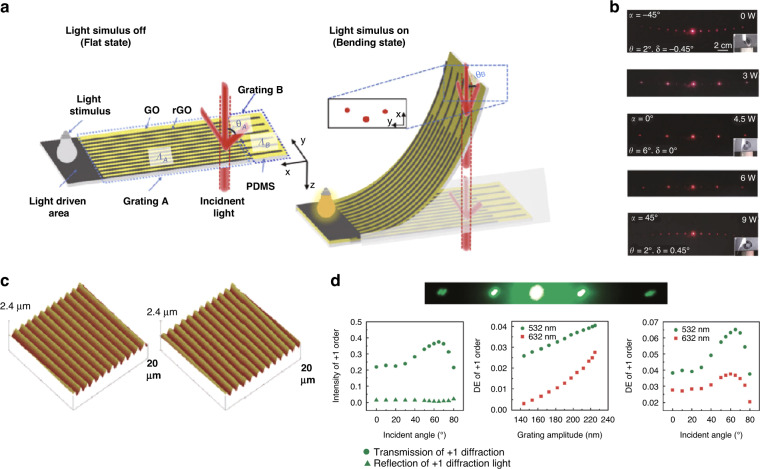
LIG-based 1D diffraction gratings. **a** LIG-based diffraction grating system using femtosecond laser direct writing. **b** Changes in the light-gathering epithelium as the diffraction grating changes with light. **c** Atomic force microscopy(AFM) image of GO-based reflective grating system. **d** Difference in diffraction of light according to wavelength and incidence angle. **a**, **b** Adapted with permission from ref. ^15^, The Optical Society. **c**, **d** Adapted with permission from ref. ^7^, Royal society of chemistry (RSC)

Optical diffraction gratings have a periodical change of amplitude or phase^7,14,15^. They can be used in a wide range of applications, such as beam steering, switching, beam shaping, and spectral shaping. Planar and curved dielectrics or metals with periodic structures function as optical gratings. Recently, optical gratings patterned with a variety of methods for LIG and GO/rGO have been introduced. Figure 15a introduces a transmission-type optical grating on a flexible substrate, which was created by femtosecond laser direct writing on a GO-coated PDMS thin-film^15^. By combining the relatively large coefficient of thermal expansion (CTE) of the PDMS substrate and the low CTE of the GO/rGO layer, an intriguing grating was developed that allows the user to actively control the bending. This device is advantageous in that the geometric grating interval can be adjusted owing to its unique bending structure. (See Fig. 15b) Based on these results, LIG or GO/rGO devices could implement multiple gratings with ease. The rGO optical gratings can also be produced using a soft lithography duplication process^7^; Fig. 15c shows an optical grating fabricated thereby. The resulting reflective grating presents the performances as shown in Fig. 15d.

### Patterning of plasmonic graphene nano/microstructures

**Fig. 16:**
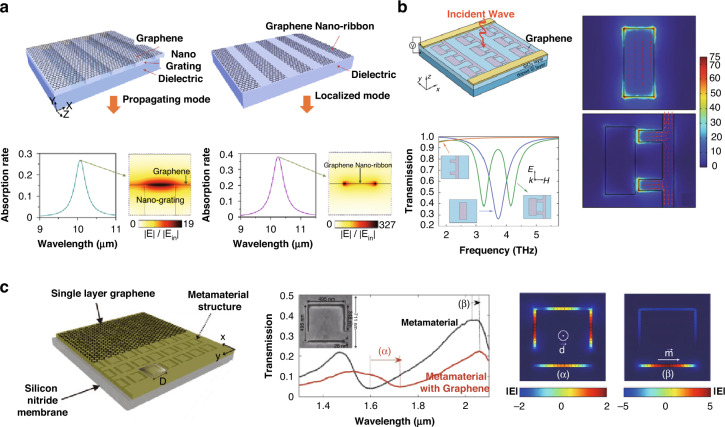
Plasmonic LIG nanostructures. **a** Schematics of the configurations for the excitations of propagating graphene plasmons (**a-1**) and localized graphene plasmons (**a-2**). The absorption curves of the propagating plasmonic mode. **b** unit cell of the graphene-based PIT metamaterial design and amplitude of electric field |*E*| and electric current density. **c** Schematic of the fabricated complementary split-ring metamaterial and electric field maps at the trapped-mode (α) and the dipole (β) resonance. **a** Adapted with permission from ref. ^113^, The Optical Society. **b** Adapted with permission from ref. ^111^, Royal society of chemistry (RSC). **c** Adapted with permission from ref. ^112^, The Optical Society

Metal nanostructures with various geometries generate surface plasmon resonance (SPR) in response to specific optical frequencies^7,111,112^. This metallic shape acts as a local antenna that can control several aspects of the interacting electromagnetic waves, including the amplitude, polarization, refraction, and changes in the direction of the reflected beam. However, it is known that the plasmonic reaction of general metals is weak in the mid-infrared (MIR) or at longer wavelengths (THz) because the interaction between electromagnetic waves and electrons is weak. Owing to its unique mechanical, electrical, and optical properties, graphene with two-dimensional (2D) carbons arranged in a hexagonal arrangement has a very high quantum efficiency for light/matter interactions, optical nonlinearity, and exhibits unique plasmonic properties. In recent years, graphene has been recognized as a new material for supporting surface plasmons in the long wavelength region (IR to THz). These graphene plasmons can be controlled via gating, doping, chemical means, and interactions with novel metals. In the following section, we describe recent research on plasmonic optics using graphene from a broad perspective. Figure 16a–c show graphene-based plasmonic nanostructures of various shapes. Figure 16a depicts two plasmonic structures with different shapes, and information about the propagation mode, the local plasmon mode, and the electric field generated by the structure^113^. In Fig. 16b, c, the numerical simulation values related to the concentration of the electric field according to the shape of the graphene-based plasmonic structure are presented. As such, graphene-based plasmonic structures are used as plasmonic materials, especially in the long wavelength band.

### Laser patterning of graphene holograms

**Fig. 17:**
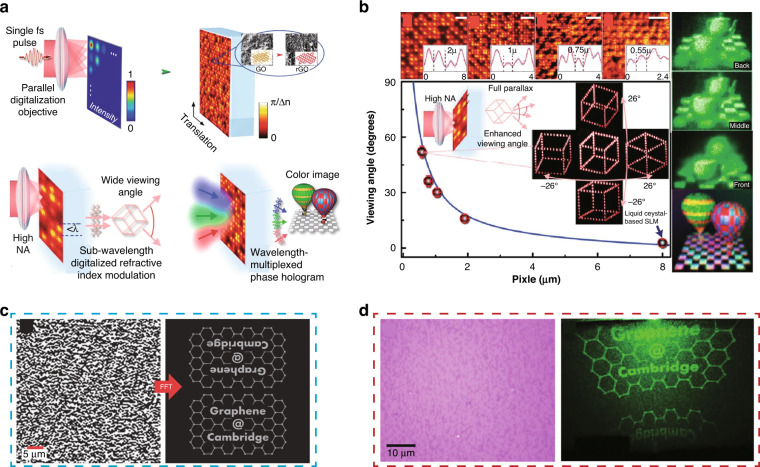
Laser patterning of graphene holograms. **a** Schematic illustration of the optical digitalization of refractive-index/phase modulation by athermal photoreduction using a single fs pulse. **b** Typical examples of microscopic images of sections of rGO holograms produced by objectives with different NA values. **c** Results for 2D graphene based holographic pattern. (d) Optical microscopy image of a patterned 3-layer graphene sample. (Left) The holographic image produced when the patterned graphene sample is illuminated with a green laser beam. (Right). **a**, **b** Adapted with permission from ref. ^9^, Springer Nature. **c**, **d** Adapted with permission from ref. ^11^, Wiley-VCH

1D graphene grating already presents that the graphene readily supports coherent diffraction. As the continued work, graphene-based ultra-thin diffraction holography was investigated using a proven diffraction effect. A graphene hologram consists of a binary, multi-phased intensity, or phase mask that performs a Fourier transform to produce an arbitrary image^9,11,52^. For example, a phase difference can be induced by gradually reduced GO layer, as shown in Fig. 17a^9^. The phase mask produced in this manner consists of multiple step heights, and the holographic image changes according to light incidence angle, as shown in Fig. 17b. The generated image can be reproduced in various ways, from a simple hexahedral shape to a complex hot-air balloon image. In addition, as shown in Fig. 17c, the intensity-based mask was designed using the Gerchberg-Saxton algorithm^11^. By irradiating the mask with a suitable light source, it is possible to obtain an arbitrary pattern, as shown in Fig. 17d. For these holographic images, it is necessary to carefully consider the generation of graphene-based patterning because the resolution of the pattern affects that of the hologram. The diffraction devices produced from various graphene materials can serve as practical demonstrations of the diffraction element.

## Ultra-thin LIG planar diffractive lens: representative applications

Traditionally, optical devices that control light propagation have been made using bulk materials with well-established specific shapes, such as prisms, convex or concave lenses/mirrors that work based on refraction or reflection. However, traditional refractive/reflective optics require a relatively long propagation length and a large working area to guide and shape the wavefront as required. As such, this requires a large volume and weight in existing optical devices that hinders system simplification and miniaturization. Recently, the concept of flexible/stretchable photonics (FP/SP) has been introduced by directly integrating or fabricating conventional photonic devices on deformable polymer substrates. In general, FP refers to optical devices that are fabricated on flexible substrates that can be mechanically deformed (e.g., bent, folded, rolled, twisted, stretched, or compressed) without compromising optical performance^3,8,53^. FPs are usually ultra-thin, light-weight, and tough to facilitate flexibility and stretching. The form factor is critical for enabling emerging applications in both consumer and industrial markets. FPs are currently integrated into equipment or products for imaging and display^3^, instrumentation^114^, energy-harvesting devices^115^, and photonic circuits^114,116^. In the following sections, we will discuss how to utilize the patterned FP/SP-based FZPs, gratings, holograms, and so on, for wide industrial applications. We will describe key optical components that utilize the unique flexibility, elasticity, miniaturization, and light-weight properties of FP/SPs, which can be applied to future industrial surface profilometry, bio-medical imaging, and outer space applications.

### Laser patterning of micro FZP array for dynamic wavefront sensing

**Fig. 18:**
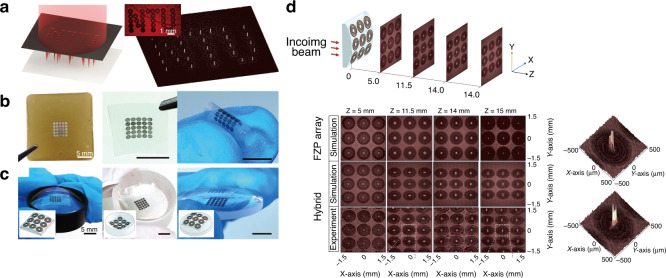
Realization of hybrid optics by transferring flexible PDL on rigid refractive optics. **a** Hybrid optics fabricated by transferring ultra-thin diffractive micro-optics array to traditional rigid refractive optics. **b** 5 × 5 lens array patterned on GO film which is coated on a cover glass and subsequently transferred onto a thin PDMS film. Scale bar: 5 mm. **c** The lens array on PDMS film conforms to various hybrid optics applications. Scale bar: 5 mm. **d** Schematic of the beam propagation from a collimated laser beam to hybrid refractive-diffractive optics and the resulting beam profiles were simulated at different focal depths. **a**–**d** Adapted with permission from ref. ^8^, Elsevier

Flexibility and stretchability are of great interest in wearable and integrated electronics and are also applicable to optoelectronic devices^3,8,53^. Another key advantage of ultra-thin GO micro-optics is that they can be easily transferred to elastomer substrates. An ultra-thin GO micro-optics array was fabricated and transferred onto a PDMS substrate to realize flexible and stretchable micro-optics functionality over a wide wavelength range. Using a simple GO coating on a given substrate and subsequent *fs* direct laser writing, a micro-diffractive lens array with arbitrary lateral patterns can be fabricated. Figure 18a shows the working principle of the ultra-thin GO lens array, patterned to the shape of ‘NTU’ on a glass substrate, a real sample image, and the focal plane captured by a CCD camera^8^. Each micro FZP had ten concentric opaque rGO rings designed to have a focal length of 15 mm at a wavelength of 638 nm; the outer diameter of each structure was 875 μm. An incident collimated laser beam was focused by each subset lens without any significant power loss or field deviation from the design, as shown in Fig. 18a. The ultra-thin microlens array was first patterned onto the GO film, which was previously drop-casted onto a glass substrate, as shown in Fig. 18b.

The patterned micro-optic array was then transferred to a flexible and stretchable substrate. These transferred rGO structures can provide a better transmission contrast than GO/rGO and have better optical characteristics at the focal plane. Figure 18b shows the fabricated 5 × 5 rGO lens array transferred from the GO/rGO film to the PDMS. The combination of PDMS and rGO facilitates high flexibility and stretchability of rGO so that its optical performance can be preserved without notable degradation even under high mechanical deformations, such as bending, rolling, and twisting. This flexibility and stretchability allow for novel wavefront shaping capability in ultra-thin diffractive micro-optics by simply attaching them to the surface of existing bulk refractive or reflective optical elements (as shown in Fig. 18c). Whereas traditional rigid optics need to be carefully aligned with many mechanical supports, stretchable thin-film micro-optics can be simply attached on top of existing optics without much loading or support. Figure 18d shows an example of hybrid optics in which a flexible rGO/PDMS diffractive FZP array with a focal length of 15 mm is combined with a bulk cylindrical lens of 50 mm focal length. The rGO microlens array component was placed directly on the cylindrical lens, and PDMS functioned as a thin flexible substrate that followed the surface of a rigid lens with uniform thickness. The propagation characteristics of the hybrid optics were confirmed by comparing the experimental results with simulations. The previously reported fabrication and performance of the flexible dielectric optics for wavefront tuning using ultra-thin nanomaterials integrated into PDMS are presented in Table 5. Among these techniques, the method described in this article is the simplest material preparation and optomechanical configuration for FZP patterning. This concept for manufacturing hybrid optics is a practical approach for combining the advantages of refractive optics, reflective optics, diffractive optics, and metasurfaces, without the need for precision manufacturing of micropatterns on top of free-form optics.

**Table 5:** Summary of fabrication methods of flexible diffractive optics and their optical performances

Lens Type	Reference	Image	FZP preparation				FZP performance		
			Thickness	Diameter	Process	Materials	Wavelength	Focal length	purpose
Meta-surface	Kamali et al.^43^		720 nm	1 mm	Electron Beam Lithography	Amorphous Silica & PDMS	915 nm	3.5 mm	Focusing
FZP	Moghimi et al.^3^		100 nm	450 μm	Lithography	Silica nanowire & PDMS	620 nm	N/A	Imaging
FZP	Li et al.^53^		10 μm	∼650 μm	Lithography	Carbon nanotube & PDMS	635 nm	7.00–8.47 mm	Focusing
FZP	Low et al.^8,139^		0.9 μm	656 μm	FsDLW with Galvano scanner	Graphene Oxide & PDMS	638 nm	15 mm	Focusing

The images used in Table 5 were adapted with permission from Springer Nature^3,43,53^, Elsevier^8^, IOP publisher^139^

By monitoring the lateral positions of the array focal spots using a CCD or CMOS camera, the fast dynamics of the incident wavefront can be measured in real-time. If the well-collimated plane wavefront is reflected at the non-planar deformed surface, the wavefront will be distorted; this distortion can be traced at high speed. This can be directly used in array confocal microscopes for high-speed industrial surface measurement with sub-micrometer measurement precision. For machining large-scale optics, such as large telescope mirrors, EUV lithography mirrors, and space optics, the surface profile should be measured firstly by a coordinate measuring machine (CMM), secondly by wavefront sensing Shack-Hartmann sensor, and finally by optical interferometry. Therein, the arbitrary-shaped micro-PDL array can expand the dynamic range and resolution of the Shack-Hartmann sensors^30–33^. This can be also used for the precision measurement of atmospheric wavefront distortions.

### Compact micro-lens for endoscopic optical coherence tomography

#### Introduction to optical coherence tomography (OCT)

**Fig. 19:**
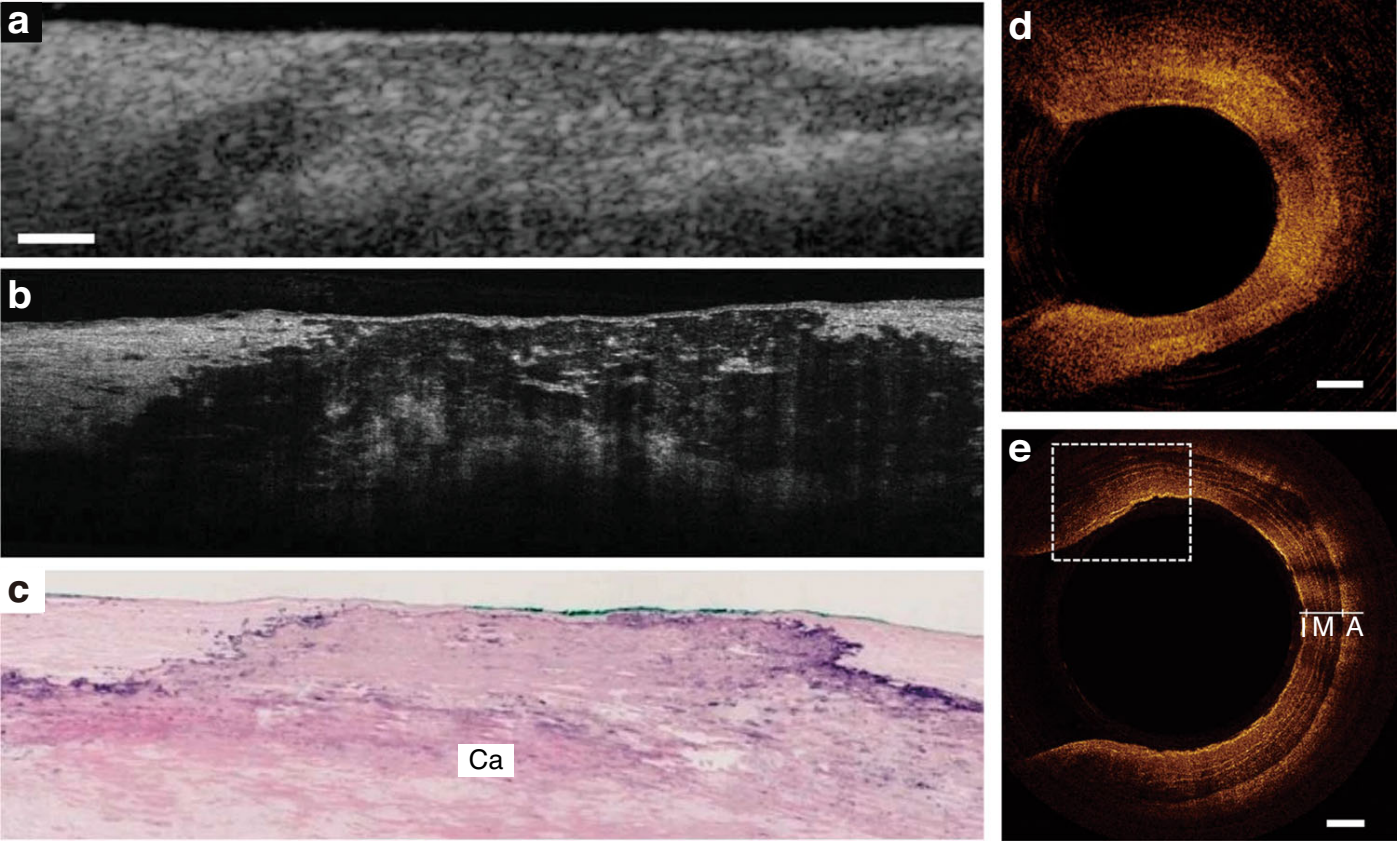
Compact PDLs for micro-OCT application. **a** OCT, (**b**) OCT and (**c**) histology images of fibrocalcific human cadaver coronary plaque. Endoscopic (**d**) OCT and (**e**) OCT images of healthy swine coronary artery. Scale bars: 200 μm. **a**–**c** Adapted with permission from ref. ^117^, Springer Nature. **d**, **e** Adapted with permission from ref. ^21^, Springer Nature

Graphene-based PDLs can be utilized in the medical field, especially in the imaging optics of endoscopic probes for OCT. OCT is a non-invasive biological imaging method that facilitates depth-resolved tomographic images by exploiting the interference of light, as shown Fig. 19^21,117,118^. Several ground-breaking studies on high-resolution OCT have shown that the microscopic structures of biological samples can be visualized at the sub-cellular level with high-speed and high-sensitivity^117^. In addition, OCT with flexible endoscopic probes enables the imaging of internal micro-structures, such as blood vessels, the esophagus, and the eustachian tubes (Fig. 19d, e)^21,119,120^.

#### Key optical parameters in OCT

**Fig. 20:**
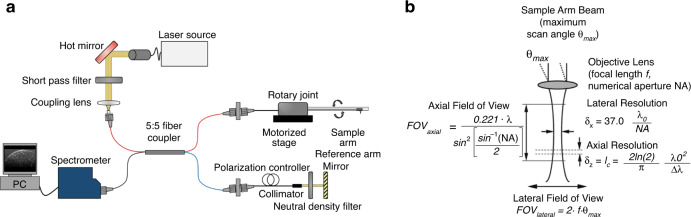
OCT: system layout. **a** Schematic illustration of an endoscopic OCT system. **b** Schematic illustration of the OCT sample arm optics. **a** Adapted with permission from ref. ^21^, Springer Nature. **b** Adapted with permission from ref. ^145^, Springer Nature

The basic principle of OCT is as follows: the beam from a light source travel through an interferometer and is divided into a sample and reference arms, through the optical components, such as a beam splitter and a fiber coupler (Fig. 20a). The backscattered light from the sample interferes with the light reflected from a reference mirror and is detected by a photodetector. Therefore, an axial OCT image is encoded in the spectral domain of the interference signal, while the lateral image is typically acquired using scanning systems based on galvo mirrors or fiber optic rotary junctions^121^. In general, the axial resolution (*Δz*), lateral resolution (*Δx*), and axial field-of-view of OCT are defined as follows (Fig. 20b)^21^:

$$\Delta{\rm {z}}=l_c=\frac{2\ln(2)}{\pi}\,\frac{\lambda^2_{0}}{\Delta\lambda},\,\Delta{\rm{x}}= 37.0\frac{{\lambda _{0}}}{{{{NA}}}},\,{{FOV}}_{{{axial}}}=\frac{0.221\lambda_{0}}{{sin}^{2}\left[\frac{{sin}^{-1}\left({{NA}}\right)}{2}\right]}$$

where *λ*_*o*_ is the center wavelength, *∆λ* is the bandwidth, and NA is the numerical aperture of the imaging optics. Therefore, to achieve high resolution in the axial and lateral directions, a light source with a short center wavelength, broad bandwidth, and high-NA imaging optics are required.

#### How can PDLs be applied future endoscopic fiber optic probes?

**Fig. 21:**
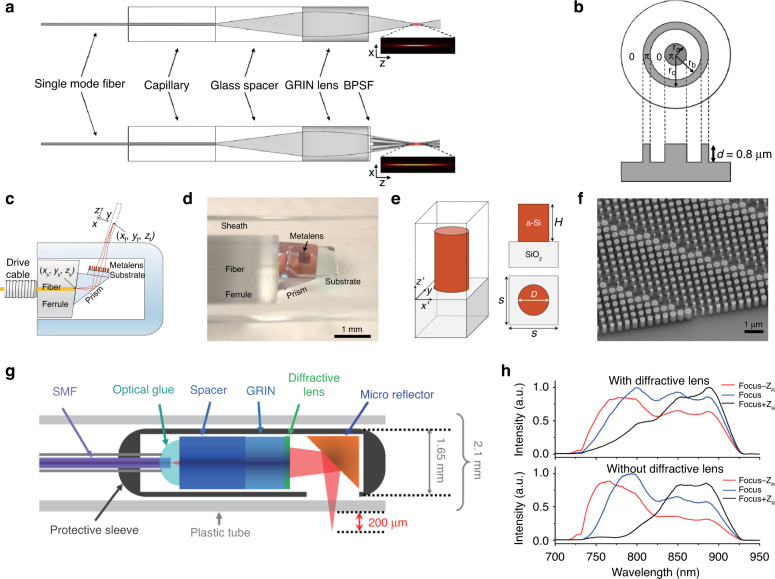
Endoscopic OCT: future development directions. **a** Schematic illustration of the endoscopic OCT probe without (top) and with (bottom) BPSF. **b** Structure of the BPSF. Adopted from. **c** Schematic illustration and (**d**) photographic image of the endoscopic μOCT probe using metalens. **e** Schematic of individual metalens building block and (**f**) scanning electron micrograph image of metalens. **g** Schematic illustration of the endoscopic μOCT probe using diffractive lens to alleviate chromatic aberration. **h** Reflected spectra of the probe with (top) and without (bottom) diffractive lens. **a**, **b** Adapted with permission from ref. ^127^, The Optical Society. **c**–**f** Adapted with permission from ref. ^131^, Springer Nature. **g**, **h** Adapted with permission from ref. ^24^, The Optical Society

For endoscopic OCT applications, several fundamental challenges must be overcome, as shown in Fig. 21. First, there is a trade-off between the lateral resolution and the probe size. The lateral resolution of an OCT system is determined by its NA, which is governed by the diameter and focal length of the imaging lens. It is known that the size of the fiber-optic probe must be at least 1–2 mm to yield a lateral resolution of 2–3 µm^21,122–126^. Such large sizes can cause critical problems when light traverses through thin or tortuous blood vessels. Several solutions have been proposed to address this problem. Li et al.^22^ developed an ultra-thin monolithic probe based on three-dimensional (3D) micro-printed optics. They printed an aberration-corrected micro-optic lens on the side of a coreless fiber to miniaturize the overall size while maintaining a high lateral resolution. Moreover, super-resolution techniques such as the use of amplitude pupil filters have been suggested to alleviate these trade-off issues^117^. If we introduce ultra-thin PDLs by DLW, we can realize any arbitrarily designed transferrable micro-optics and amplitude pupils; this can readily support this first trade-off.

Second, the trade-off between the lateral resolution and axial field of view must be handled. The use of a lens with a higher NA for better lateral resolution reduces the axis field of view. This can cause imaging difficulties when the probe is not centered within a vessel or the vessel’s shape, or size is irregular. Tan et al.^23^ fabricated a conical-tip fiber using a selective chemical etching technique to improve the axial imaging depth. Xing et al.^127^ and Kim et al.^21^ applied a binary-phase spatial filter (BPSF) to the front of the distal end of a probe to extend the depth of focus in the axial direction. The BPSF was designed using a customized optimization algorithm and fabricated using soft lithography techniques. Yin et al.^124^ generated a coaxially focused multimode (CAFM) beam by inserting a multimode fiber immediately after a single-mode fiber. It has been reported that this self-imaging wavefront division optical system extends the depth of focus by a factor of five compared to a normal Gaussian beam. If we introduce ultra-thin PDLs by DLW, we can support the OCT performance in terms of lateral resolution and axial field-of-view on demands.

Third, the cylindrical transparent sheath that protects the imaging probe from blood and other foreign substances causes astigmatism. This can degrade the image quality, especially with regard to the lateral resolution and the signal-to-noise ratio (SNR). An asymmetric ball lens is generally used to overcome astigmatism. However, given that it is difficult to precisely fabricate a ball lens with a high NA, cylindrical reflectors have also been used^124,128,129^. Lee et al.^130^ reported on a cost-effective method for overcoming astigmatism. It has been reported that astigmatism can be corrected by controlling the curvature radius of the epoxy window. Pahlevaninezhad et al.^131^ calibrated astigmatism and nonchromatic aberration using a precisely designed metalens-based imaging probe. When we introduce ultra-thin PDLs by DLW, non-symmetric optical aberrations induced by the sheath can be simply compensated for during the design process, which can provide a better-quality 3D focus in micro-endoscopic OCT.

Finally, the broad-spectrum bandwidth of nearly 300 nm in high-resolution OCT causes severe chromatic aberration. Uncorrected chromatic aberration causes each wavelength sections to be focused at different axial positions, which degrades the axial and longitudinal resolution. Yuan et al.^132^ utilized a fiber-optic ball lens instead of a regular GRIN lens to effectively correct for chromatic aberration. In addition, Xi et al.^24^ alleviated chromatic aberration using a diffractive lens with negative chromatic dispersion. When we apply ultra-thin PDLs by DLW, one can make different radial sections to work with different wavelength regimes so that we can actively tailor and compensate the chromatic aberrations in micro-endoscopic OCT.

### Ultra-thin PDLs for future light-weight space optics

Graphene-based materials could be applied in future space missions, in which spacecraft acquire images in various orbits. Reconnaissance satellites obtain visible light images of specific regions on Earth and space telescopes therein acquire the images of celestial bodies at various wavelengths. To obtain high-resolution images, the diameter of the primary optics should be increased. However, conventional refractive and reflective optics with a large aperture are generally heavy, so there have been limitations in resolution improvement. The satellite’s launching cost increases proportionally to the weight of the satellite payloads. In addition, as the primary optics gets larger, numerous actuators are included for adaptive control in order to prevent the gravitational deformation induced by the mass of the optics^25,73^. Therefore, extensive research works have been performed to construct light-weight optics for future space missions^25^.

#### Key advantages of PDL in future space optics

**Fig. 22:**
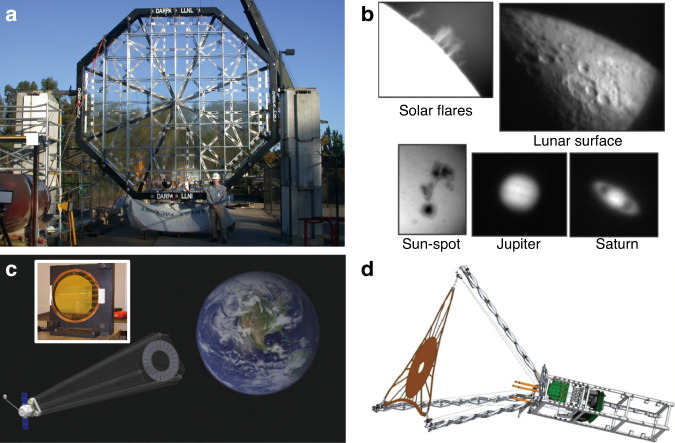
PDLs for future space optics. **a** 5 m Fresnel lens made with 700 mm glass. **b** Optical performance for LLNL color-corrected diffractive telescopes. **c** Notional MOIRE spacecraft (inset: Fresnel phase plate). **d** FalconSAT-7 deployed configuration. **a** Adapted with permission from ref. ^134^, SPIE. **b** Adapted with permission from ref. ^27^, SPIE. **c** Adapted with permission from ref. ^135^, SPIE. **d** Adapted with permission from ref. ^137^, SPIE

The introduction of PDL in space optics allows for larger aperture primary optics with a lower mass, which enables a significant reduction of the launch cost (Fig. 22). For example, the Hubble space telescope has a primary lens of 200 kgm^**−**2^, whereas the James Webb space telescope lens is reduced to 15 kgm^**−**2^. A Fresnel diffractive lens of diameter 25 m is expected to require only 10 kg and 0.02 kgm^**−**2^ ^133^. However, the capabilities of launching vehicles are limited. A folding/unfolding method was proposed and tested for the deployability of large diffractive optics^26^. Conventional reflective optics also have limitations in terms of their surface tolerance. Diffractive optics are advantageous in those points because the transmissive optics are inherently less sensitive to surface errors, and thus have low surface tolerances^134^.

#### Precedent examples of diffraction optics in space

‘Eyeglass’ is a space telescope that applies diffractive optics to the primary lens or magnifying glasses. To overcome chromatic dispersion in diffractive optics, ‘Eyeglass’ introduced an additional secondary corrector in a separate space vehicle (Fig. 22a, b)^27^. The Earth observation satellite, ‘MOIRE’, consists of three elongated structures in its body and first-order diffractive optics supported by the rear optics, as shown in Fig. 22c. The primary lens was initially folded for deployability owing to its large diameter of 5.0 m^135,136^. FalconSAT-7 is a membrane solar telescope mounted on a 3U CubeSat in a low Earth orbit. The primary diffractive optics of this telescope is a 0.2 m sheet called a photon sieve, on which billions of tiny circular dimples are etched (Fig. 22d)^137^.

#### Resistance test of LIG optics in a space environment

Given that the space environment is harsh owing to high-energy radiation, extreme temperature cycles, ultrahigh vacuum, and active oxygen, the stability of LIG should be tested. Cao et al. examined the surface morphology and focusing performance of rGO PDLs in a low-Earth orbit environment^138^. It showed that the focusing performance was well-maintained without degradation under space environment. However, active oxygen gradually deteriorated the performance of rGO as the mission period passes by. This implies that additional material protection should be carefully considered for ultra-thin PDL space optics. Basically, graphene is a very stable material; however, once some defects are generated it could propagate to the nearby area. Therefore, those possibilities should be considered and tested in the early stage ground tests. In addition, all the kinds of ground tests should be conducted considering the space mission details; the space test includes vibration tests, thermal-vacuum tests, and space radiation tests.

## Summary and future prospects

We introduced the technological trends and latest research works on ultra-thin, compact, light-weight planar diffractive lens (PDL) made of laser-induced-graphene (LIG) patterned by direct laser writing (DLW) with high design flexibility and high conformability (flexibility and stretchability). Novel hybrid optics could be realized with the aid of LIG PDLs patterned by DLW; the key advantages of refractive, reflective, and diffractive optics could be integrated into the hybrid optics for realizing future endoscopic brain imaging, high-speed space internet, industrial high-speed surface profilometry, and multi-functional mobile phones. Multi-functional asymmetric PDL arrays will also open new market chances in industries. In order to endow these new possibilities with a short lead time, in-depth understanding of the base materials (such as graphene, MoS_2_, MXene), flexible/stretchable substrates (such as PDMS, ecoflex), and photo-material interaction is a prerequisite. Furthermore, detailed parametric studies, multi-physics simulations, chemical characterization, molecular simulations, and interdisciplinary discussions should be accompanied.

For the real-world application of LIG PDLs, we must understand the market needs, lead the technological trends, and prepare for commercialization. There are a series of potential markets for PDLs as presented in this review, but it is challenging to expect which one will be open first. Therefore, active collaboration with industrial partners is also one of the key requirements. Interdisciplinary collaboration is also important because there could be a technological gap between the technological demands and available DLW outputs. For example, if one group solely works on DLW but not with application ideas on endoscopic bio-imaging, lightweight space optics, and complex functional hybrid optics in extended reality (XR) industries, they could lose good chances for PDLs to be applied to the real world. Therefore, the ‘Industry Academia Consortium (IAC)’ could be a good starting base for the industrialization of PDLs. The technological trends on PDLs and DLW can be monitored by academic researchers, while the market trends are traced, opened or even led by the industrial partners. Organizing and continuing the consortium could require extra efforts but it should be worthwhile if we could open new vistas of PDL and DLW. To date, most of the research works have focused on demonstrating the new functions and possibilities of LIG PDLs, not the mass production. Therefore, additional works on optimizing the manufacturing process in terms of the productivity must be followed subsequently with the industries at the commercialization final stage.
